# Plasma polyunsaturated fatty acids and mental disorders in adolescence and early adulthood: cross-sectional and longitudinal associations in a general population cohort

**DOI:** 10.1038/s41398-021-01425-4

**Published:** 2021-05-31

**Authors:** David Mongan, Colm Healy, Hannah J. Jones, Stan Zammit, Mary Cannon, David R. Cotter

**Affiliations:** 1 Department of Psychiatry, Royal College of Surgeons in Ireland, Dublin, Ireland; 2 MRC Integrative Epidemiology Unit at the University of Bristol, Bristol, UK; 3 Centre for Academic Mental Health, Population Health Sciences, Bristol Medical School, University of Bristol, Bristol, UK; 4 National Institute for Health Research Bristol Biomedical Research Centre, University Hospitals Bristol and Weston NHS Foundation Trust, University of Bristol, Bristol, UK; 5 Division of Psychological Medicine and Clinical Neurosciences, MRC Centre for Neuropsychiatric Genetics and Genomics, Cardiff University, Cardiff, UK

**Keywords:** Molecular neuroscience, Schizophrenia, Depression

## Abstract

Polyunsaturated fatty acids (PUFAs) may be pertinent to the development of mental disorders, for example via modulation of inflammation and synaptogenesis. We wished to examine cross-sectional and longitudinal associations between PUFAs and mental disorders in a large cohort of young people. Participants in the Avon Longitudinal Study of Parents and Children were interviewed and provided blood samples at two sampling periods when approximately 17 and 24 years old. Plasma PUFA measures (total omega-6 [n-6], total omega-3 [n-3], n-6:n-3 ratio and docosahexaenoic acid [DHA] percentage of total fatty acids) were assessed using nuclear magnetic resonance spectroscopy. Cross-sectional and longitudinal associations between standardised PUFA measures and three mental disorders (psychotic disorder, moderate/severe depressive disorder and generalised anxiety disorder [GAD]) were measured by logistic regression, adjusting for age, sex, body mass index and cigarette smoking. There was little evidence of cross-sectional associations between PUFA measures and mental disorders at age 17. At age 24, the n-6:n-3 ratio was positively associated with psychotic disorder, depressive disorder and GAD, while DHA was inversely associated with psychotic disorder. In longitudinal analyses, there was evidence of an inverse association between DHA at age 17 and incident psychotic disorder at age 24 (adjusted odds ratio 0.44, 95% confidence interval 0.22–0.87) with little such evidence for depressive disorder or GAD. There was little evidence for associations between change in PUFA measures from 17 to 24 years and incident mental disorders at 24 years. These findings provide support for associations between PUFAs and mental disorders in early adulthood, and in particular, for DHA in adolescence in relation to prevention of psychosis.

## Introduction

Fatty acids are the major metabolic products of lipid metabolism. They are categorised by number of carbon-carbon double bonds: saturated fatty acids have none, monounsaturated fatty acids have one and polyunsaturated fatty acids (PUFAs) have two or more. PUFAs are further subdivided by the position of the first carbon-carbon double bond from the methyl end of the molecule. This occurs at the sixth carbon atom in omega-6 PUFAs and the third in omega-3 PUFAs. Omega-6 (n-6) PUFAs, such as linoleic acid and arachidonic acid, produce n-6 eicosanoids which have broadly pro-inflammatory effects. Omega-3 (n-3) PUFAs, including alpha-linolenic acid (ALA), eicosapentaenoic acid (EPA) and docosahexaenoic acid (DHA), produce n-3 eicosanoids which are generally anti-inflammatory^1^. Alpha-linolenic acid cannot be synthesised by humans and thus must be obtained directly from dietary intake. Although humans can convert ALA into EPA and DHA, the efficiency of this conversion is limited and thus EPA and DHA must also be obtained from the diet to maintain adequate levels^2^. Dietary sources of ALA include flaxseed and soybean oils, while EPA and DHA are found in oily fish or supplements.

Humans may have evolved on a diet with a relatively balanced ratio of n-6:n-3 PUFAs, but in the average modern Western diet this ratio may be 10:1 or higher^3^. Given the broadly opposing pro- and anti-inflammatory effects of their lipid mediators^1^, the balance of n-6 and n-3 fatty acids is thought to be relevant to disease states characterised by inflammation such as cardiovascular disease^4^. There is a body of evidence implicating low-grade inflammation in association with the presence or risk of mental disorders such as schizophrenia and depression, at least in a subset of affected patients^5,6^. The precise biological mechanisms by which inflammation may contribute to the development of mental disorders are complex, but may include modulation of hypothalamic-pituitary-adrenal axis activity, neurotransmission, neurodegeneration and microglial activation^7,8^. A lower ratio of n-6 to n-3 fatty acids may be associated with reduction in inflammation^4^ and thus presents one possible mechanism by which dietary factors can modulate inflammatory processes.

Aside from their effects on inflammation, PUFAs have several other biological actions which may be of relevance to mental disorders. For example, through reorganisation of lipid rafts (clusters of membrane proteins and receptors that contribute to signal transduction), n-3 PUFAs can modulate intracellular and intercellular signalling, cytokine secretion and immune activation^9,10^. n-3 PUFAs also contribute to the structural integrity and fluidity of neuronal membranes^11^. Of the n-3 PUFAs, DHA is particularly important in the brain, where it comprises ~90% of n-3 PUFA content and 10–20% of the brain’s total lipids^12^. In addition to its neuronal membrane effects, DHA may protect against oxidative damage^13^ and promote neurite growth and synaptogenesis^14^.

Observational studies provide evidence of decreased n-3 PUFA levels in patients with schizophrenia^15^, depressive^16^ and anxiety disorders^17^ relative to controls. PUFA deficiency has also been implicated in conditions such as attention deficit hyperactivity disorder^18^, autism spectrum disorder^19^ and Alzheimer’s disease^20^. It is however unclear whether PUFA abnormalities precede the onset of mental disorders. In addition, factors such as body mass index (BMI), smoking and socio-economic status (which may be associated with both dietary quality and, independently, mental disorders) may confound observed associations^21–23^. In clinical trials, n-3 supplementation for treatment or prevention of mental disorders has produced mixed results^24^. For example, in individuals at clinical high-risk of psychosis, n-3 supplementation initially showed beneficial effects in reducing transition to psychotic disorder^25^. This finding was not replicated in a subsequent trial^26^, although on further analysis several PUFA measures were associated with severity of psychopathology^27,28^. Associations between PUFAs and mental disorders in adolescence and early adulthood have not been extensively studied, even though most mental disorders have their onset during this critical period of development^29^.

We sought to address three main questions regarding temporal associations between plasma PUFA measures and three mental disorders (psychotic disorder, depressive disorder and generalised anxiety disorder) in adolescence and early adulthood: (1) Are PUFAs associated with these disorders cross-sectionally at age 17 years? (2) Are PUFAs associated with these disorders cross-sectionally at age 24 years? (3) Are PUFAs at age 17 years associated with these disorders longitudinally at age 24 years?

## Methods

### Participants and study design

The Avon Longitudinal Study of Parents and Children (ALSPAC) is a prospective birth cohort study^30–32^. The study website contains details of all data available through a fully searchable data dictionary and variable search tool (http://www.bristol.ac.uk/alspac/researchers/access). Pregnant women in Avon, UK with expected delivery dates between 1st April 1991 and 31st December 1992 were invited to participate. A total of 14,541 pregnancies were enroled with 13,988 children alive at 1 year of age. When the oldest children were ~7 years old, an attempt was made to bolster the initial sample with eligible cases who did not join originally. The total sample size for data from age 7 is 15,454 pregnancies with 14,901 children alive at 1 year of age. Data were collected and managed using REDCap electronic data capture tools^33,34^.

Participants were invited to attend clinics at multiple timepoints where anthropometric measurements, questionnaires, interviews and blood sample collection were completed. The current study was based primarily on data from two such clinics: first when the participants were approximately 17 years old, and subsequently when they were approximately 24 years old.

### Exposures

Participants were requested to fast overnight or for at least 6 h prior to attendance at both the age 17 and age 24 clinics. Blood samples were collected according to a standardised protocol. Samples were obtained between 0800 hours and 14 hours in > 99% of cases at both clinics. Following collection, samples were centrifuged and stored at −80 °C until analysis. For the age 17 clinic, the range of storage time between plasma collection and analysis was 2.4–5.1 years. For the age 24 clinic, this range was 0.3–2.7 years.

Summary and specific fatty acid plasma levels were measured using a high-throughput nuclear magnetic resonance spectroscopy platform^35^. We considered four PUFA measures as exposures in the current study: total n-6 fatty acids (mmol/l); total n-3 fatty acids (mmol/l); n-6:n-3 fatty acid ratio; and DHA expressed as percentage of total fatty acids.

### Outcomes

We examined three non-mutually exclusive outcomes.

#### Psychotic disorder

At the age 17 and age 24 clinics, participants completed the semi-structured Psychosis-Like Symptoms Interview (PLIKSi) to assess for psychotic experiences^36^. The PLIKSi asks 12 core questions regarding psychotic experiences comprising hallucinations, delusions and experiences of thought interference. Participants who answered ‘yes’ or ‘maybe’ to stem questions were cross-questioned to establish whether the experiences were psychotic, and these were coded according to the Schedules for Clinical Assessment in Neuropsychiatry^37^. Trained interviewers rated symptoms as ‘not present’, ‘suspected’ or ‘definite’ and coded whether the experience was attributable to sleep or fever. In line with previous studies from this sample^38,39^, psychotic disorder was defined as having at least one definite psychotic experience not attributable to sleep or fever which recurred at least once per month over the previous six months, and was associated with severe distress, marked impairment of the participant’s social or occupational functioning, or led them to seek help from a professional source.

#### Depressive disorder

At the age 17 and age 24 clinics, participants completed the self-administered computerised version of the Clinical Interview Schedule Revised (CIS-R)^40^. The CIS-R includes questions about the recent occurrence, severity, duration and onset of depressive symptoms providing diagnoses of mild, moderate or severe depressive disorder according to the International Classification of Diseases version 10 (ICD-10)^41^. In the current study, this outcome was defined as meeting ICD-10 criteria for moderate or severe depressive disorder.

#### Generalised anxiety disorder (GAD)

The CIS-R also contains questions pertaining to anxiety symptoms to determine diagnosis of GAD according to ICD-10 criteria^41^. In the current study, this outcome was defined as meeting ICD-10 criteria for GAD.

### Confounders

Based on a systematic review of non-dietary factors associated with n-3 PUFA levels^42^ we considered the following as a minimal set of confounders: precise age, sex, BMI and cigarette smoking (average number of cigarettes smoked per day in preceding 30 days).

With respect to other candidate confounders, we examined the following additional characteristics contemporaneous with exposures: alcohol intake as measured by Alcohol Use Disorders Identification Test (AUDIT) score^43^ at 17 years and AUDIT-Concise (AUDIT-C) score^44^ at 24 years; and regular cannabis use (defined as using cannabis more than once monthly at 17 years, and weekly or daily use at 24 years).

We examined the following prenatal socio-economic factors: family home ownership status during pregnancy (own home or mortgage/rented or other) as measured by a questionnaire completed by mothers at 8 weeks gestation; highest maternal educational qualification (less than O-level/ O-level or higher) as measured by a questionnaire completed by mothers at 32 weeks gestation; and parental social class based on occupation (manual/non-manual) of the participant’s mother or father (whichever was highest) as measured by a questionnaire completed by mothers at 32 weeks gestation.

Finally, we also examined the following characteristics in childhood: average take-home family income as measured by a questionnaire completed by mothers when the participant was approximately 8 years old (<£400 per week/≥£400 per week); and IQ as measured by the Wechsler Intelligence Scale for Children (Third Edition)^45^ at age 8 years.

Further information regarding the selection of confounding variables is provided in Supplementary Methods.

### Statistical analyses

Statistical analyses were performed in Stata 16 (StataCorp).

#### Descriptive analyses

We compared sociodemographic characteristics in participants who attended either the age 17 or age 24 clinics versus the rest of the ALSPAC cohort. We also compared characteristics at both 17 and 24 years according to whether PUFA data were available. Finally, we compared characteristics at both ages according to whether participants met criteria for at least one mental disorder, and stratified by type of disorder.

Categorical variables were summarised using proportions and compared using chi-squared tests. Continuous variables were summarised using the mean and standard deviation or median and interquartile range and compared using two-tailed *t*-tests (with unequal variances specified where indicated by Levene’s test *p* < 0.05) or Mann–Whitney U tests as appropriate.

#### Multiple imputation

To address potential bias arising from missing data, we imputed missing data for exposures and confounder variables for participants with outcomes data available. Fifty imputed datasets were generated using multiple imputation with chained equations. In addition to the outcomes, exposures and confounders above, several auxiliary variables were included in the imputation model to make the missing-at-random assumption more plausible. Further details are provided in Supplementary Methods.

#### Logistic regression analyses

Logistic regression was used to determine associations between exposures (PUFA measures) and outcomes (mental disorders) cross-sectionally at 17 years; cross-sectionally at 24 years; and longitudinally from 17 to 24 years. Two sets of models were used to evaluate longitudinal associations: firstly with PUFA measures at 17 years as the exposure variable, and secondly with change in PUFA measures (PUFA measure at 24 years minus PUFA measure at 17 years) as the exposure variable. Participants who met criteria for the relevant outcome at 17 years were excluded from longitudinal models to enable analysis of incident disorders in early adulthood. Estimates were combined across imputed datasets using Rubin’s rules. All PUFA measures were transformed to z-scores such that odds ratios (ORs) derived from logistic regression models may be interpreted per standard deviation increase in PUFA measure.

#### Confounders

We first assessed crude associations in models that were unadjusted for confounders. The minimum set of confounders (age, sex, BMI and average number of cigarettes smoked per day measured contemporaneously with exposures) were then included as covariates in adjusted models, which were considered as the base models. Longitudinal models examining change in PUFA measures were adjusted for sex, change in BMI (BMI at 24 years minus BMI at 17 years) and change in average number of cigarettes smoked per day (number of cigarettes at 24 years minus number of cigarettes at 17 years). Finally, in sensitivity analyses, the remaining candidate confounders (AUDIT score at 17 years, regular cannabis use at 17 years, homeownership at 8 weeks gestation, parental social class at 32 weeks gestation, family income at 8 years and IQ at 8 years) were included as covariates one at a time and the effect estimates compared to those for the base models to assess for evidence of further potential confounding.

### Ethical approval and consent

Ethical approval for the ALSPAC study was obtained from ALSPAC Ethics and Law Committee and local research ethics committees. Consent for biological samples was collected in accordance with the Human Tissue Act (2004). Informed consent for use of questionnaire and clinic data was obtained following recommendations of the ALSPAC Ethics and Law Committee at the time.

## Results

### Sample characteristics and descriptive analyses

Sample derivation is summarised in Supplementary Fig 1.

#### Characteristics of participants according to clinic attendance

A total of 9919 participants were invited to the age 17 clinic of whom 5215 (52.6%) attended. In total 9958 participants were invited to the age 24 clinic of whom 4019 (40.4%) attended. Participants who attended one or both clinics (*n* = 6087) differed compared to the rest of the initially-recruited ALSPAC sample on multiple socio-demographic characteristics (Supplementary Table 1).

#### Characteristics of participants according to PUFA data availability

Of 5215 participants who attended the age 17 clinic, 3167 (60.7%) had PUFA data available. One participant with clearly outlying PUFA data was excluded, resulting in 3166 participants. Of 4019 participants who attended the age 24 clinic, 3257 (81.0%) had PUFA data available.

There was evidence of differences on several characteristics between participants with and without PUFA data available at 17 years (Supplementary Table 2) and at 24 years (Supplementary Table 3) including precise age, sex, BMI, alcohol intake, smoking status, socio-economic factors, childhood IQ and presence of moderate/severe depressive disorder.

#### Characteristics of participants according to mental disorder

At 17 years, 79 of 4718 participants assessed (1.7%) met criteria for psychotic disorder; 227 of 4563 (5.0%) met criteria for moderate/severe depressive disorder; and 263 of 4563 (5.8%) met criteria for GAD.

At 24 years, 47 of 3889 participants assessed (1.2%) met criteria for psychotic disorder; 304 of 3966 (7.7%) met criteria for moderate/severe depressive disorder; and 386 of 3957 (9.8%) met criteria for GAD.

Among participants assessed at both 17 and 24 years, 2774 participants did not meet criteria for psychotic disorder at age 17 of which 20 (0.7%) subsequently met criteria for psychotic disorder at age 24. 2662 participants did not meet criteria for moderate/severe depressive disorder at age 17, of which 157 (5.9%) subsequently met criteria for moderate/severe depressive disorder at age 24. 2628 participants did not meet criteria for GAD at age 17, of which 205 (7.8%) subsequently met criteria for GAD at age 24.

There was evidence of differences between participants who met criteria for one or more of these disorders compared to those who did not for several characteristics at both age 17 and age 24 (Table 1) including precise age, sex, BMI, smoking status, alcohol intake, regular cannabis use, and prenatal socio-economic factors. Sample characteristics are further stratified according to specific disorder at age 17 years in Supplementary Table 4 and at age 24 years in Supplementary Table 5.

**Table 1:** Characteristics of age 17 and age 24 samples according to mental disorder status.

		Met criteria for psychotic disorder, moderate/severe depressive disorder or generalised anxiety disorder at age 17				Met criteria for psychotic disorder, moderate/severe depressive disorder or generalised anxiety disorder at age 24			
		Yes *n* = 444	No *n* = 4490	*p*	Missing data, *n* (%)	Yes *n* = 549	No *n* = 3459	*p*	Missing data, *n* (%)
Age in years, mean (SD)		17.8 (0.4)	17.8 (0.4)	0.303	0 (0%)	24.1 (0.8)	24.0 (0.8)	0.015	0 (0%)
Sex^a^, *n* (%)	Female	≥74.9%	≥54.7%	<0.001	≤0.2%^a^	≥75.1%	≥60.6%	<0.001	≤0.2%^a^
	Male	≤25.1%	≤45.3%			≤25.0%	≤39.4%		
Ethnicity, *n* (%)	White	374 (94.7%)	3846 (95.7%)	0.349	520 (10.5%)	460 (95.6%)	2954 (96.0%)	0.678	451 (11.3%)
	Non-white	21 (5.3%)	173 (4.3%)			21 (4.4%)	122 (4.0%)		
BMI in kg/m^2^, mean (SD)		23.1 (4.8)	22.8 (4.1)	0.244	100 (2.0%)	25.7 (6.3)	24.8 (4.8)	0.002	42 (1.0%)
Daily smoker, *n* (%)	No	278 (76.2%)	3385 (88.7%)	<0.001	753 (15.3%)	428 (78.4%)	3039 (89.1%)	<0.001	51 (1.3%)
	Yes	87 (23.8%)	431 yes (11.3%)			118 (21.6%)	372 (10.9%)		
Average number of cigarettes smoked per day if daily smoker, median (IQR)		10 (10)	10(8)	0.288	753 (15.3%)	10(7)	9(7)	0.447	51 (1.3%)
AUDIT score, median (IQR)		8 (7)	7(6)	<0.001	1083 (21.9%)	N/A	N/A	N/A	N/A
AUDIT-C score, median (IQR)		N/A	N/A	N/A	N/A	5(4)	5(3)	0.068	79 (2.0%)
Regular cannabis use^b^, *n* (%)	No	307 (85.5%)	3529 (93.5%)	<0.001	801 (16.2%)	491 (89.6%)	3238 (94.4%)	<0.001	31 (0.8%)
	Yes	52 (14.5%)	245 (6.5%)			57 (10.4%)	191 (5.6%)		
Home ownership status at 8 weeks gestation, *n* (%)	Own or mortgage	315 (80.4%)	3496 (87.7%)	<0.001	554 (11.2%)	385 (81.2%)	2688 (88.7%)	<0.001	504 (12.6%)
	Rent or other	77 (19.6%)	492 (12.3%)			89 (18.8%)	342 (11.3%)		
Highest parental social class at 32 weeks gestation, *n* (%)	Non-manual	324 (85.9%)	3420 (87.3%)	0.283	639 (13.0%)	395 (85.0%)	2662 (88.9%)	0.013	549 (13.7%)
	Manual	53 (14.1%)	498 (12.7%)			70 (15.0%)	332 (11.1%)		
Highest maternal educational qualification at 32 weeks gestation, *n* (%)	O-level or higher	323 (80.0%)	3318 (81.4%)	0.488	452 (9.2%)	403 (83.0%)	2585 (83.1%)	0.578	406 (10.1%)
	Less than O-level	81 (20.0%)	760 (18.6%)			88 (17.0%)	526 (16.9%)		
Take-home family income at age 8 years, *n* (%)	£400+ per week	158 (52.2%)	1874 (57.0%)	0.102	1344 (27.2%)	200 (53.8%)	1466 (59.0%)	0.056	1151 (28.7%)
	<£400 per week	145 (47.8%)	1413 (43.0%)			172 (46.2%)	1019 (41.0%)		
IQ score at age 8 years, mean (SD)		106.2 (16.1)	107.2 (16.3)	0.255	852 (17.3%)	107.4 (15.9)	108.0 (15.9)	0.461	797 (19.9%)

*AUDIT* Alcohol Use Disorders Identification Test, *BMI* body mass index, *IQR* interquartile range, *PUFA* polyunsaturated fatty acid, *SD* standard deviation, *N/A* not applicable.

^a^Exact numbers suppressed due to small cell counts for missing data.

^b^Regular cannabis use was defined as using cannabis more than once monthly at age 17 and weekly or daily use at age 24.

### Logistic regression analyses

#### Cross-sectional associations between PUFAs and mental disorders at 17 years

**Fig. 1:**
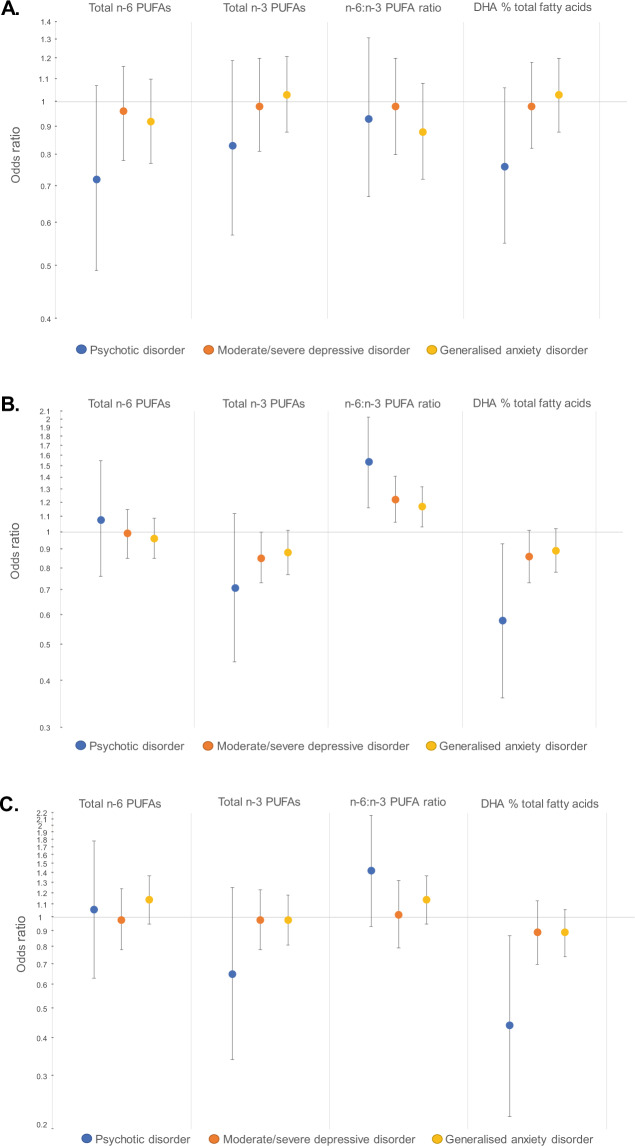
Odds ratios for associations between plasma polyunsaturated fatty acid measures and mental disorders. **A** Cross-sectional association at age 17 years. **B** Cross-sectional associations at age 24 years. **C** Longitudinal associations between PUFA measures at age 17 years and incident mental disorders at age 24 years.Data are presented on logarithmic scale. Odds ratios are per standard deviation increase in PUFA measure and adjusted for precise age, sex, body mass index and average number of cigarettes smoked per day. Error bars are 95% confidence intervals.DHA: docosahexaenoic acid; n-6: omega-6; n-3: omega-3; PUFA: polyunsaturated fatty acid.

Results from logistic regression analyses examining associations between PUFA measures and mental disorders at 17 years are presented in Table 2 and Fig. 1A. There was little evidence of associations between PUFA measures and psychotic disorder, moderate/severe depressive disorder or GAD at 17 years in crude or adjusted models.

**Table 2:** Cross-sectional associations between plasma polyunsaturated fatty acid (PUFA) measures and odds of mental disorders at 17 years.

Outcome (*n* with vs. without outcome)	Exposure	Crude models			Adjusted models		
		Odds ratio	95% CI	*p*	Odds ratio	95% CI	*p*
Psychotic disorder (79 vs. 4639)	Total n-6 PUFAs	0.90	0.65–1.26	0.544	0.72	0.49–1.07	0.107
	Total n-3 PUFAs	0.86	0.60–1.23	0.396	0.83	0.57–1.19	0.305
	n-6:n-3 ratio	1.04	0.76–1.44	0.798	0.93	0.67–1.31	0.688
	DHA % total fatty acids	0.78	0.57–1.07	0.125	0.76	0.55–1.06	0.112
Moderate/severe depressive disorder (227 vs. 4336)	Total n-6 PUFAs	1.13	0.95–1.34	0.161	0.96	0.78–1.16	0.648
	Total n-3 PUFAs	1.03	0.85–1.24	0.785	0.98	0.81–1.20	0.861
	n-6:n-3 ratio	1.07	0.88–1.30	0.497	0.98	0.80–1.20	0.815
	DHA % total fatty acids	0.99	0.83–1.18	0.918	0.98	0.82–1.18	0.845
Generalised anxiety disorder (263 vs. 4300)	Total n-6 PUFAs	1.07	0.92–1.25	0.373	0.92	0.77–1.10	0.347
	Total n-3 PUFAs	1.08	0.93–1.26	0.316	1.03	0.88–1.21	0.691
	n-6:n-3 ratio	0.95	0.77–1.16	0.582	0.88	0.72–1.08	0.227
	DHA % total fatty acids	1.08	0.93–1.25	0.299	1.03	0.88–1.20	0.731

BMI: body mass index; CI: confidence interval; DHA: docosahexaenoic acid; n-6: omega-6; n-3: omega-3; PUFA: polyunsaturated fatty acid.

PUFA measures were converted to z-scores such that the odds ratios may be interpreted as per standard deviation increase in the PUFA measure at 17 years. Covariates in adjusted models included age at attendance at age 17 clinic, sex, BMI at 17 years and average number of cigarettes smoked per day at 17 years.

#### Cross-sectional associations between PUFAs and mental disorders at 24 years

Results from logistic regression analyses examining associations between PUFA measures and mental disorders at 24 years are presented in Table 3 and Fig. 1B. There was little evidence of associations between total n-6 or n-3 PUFAs and mental disorders at age 24. However, the n-6:n-3 ratio was positively associated with all three outcomes, with the strongest evidence for psychotic disorder (adjusted OR 1.54, 95% CI 1.16–2.03). There was evidence of an inverse association between DHA % of total fatty acids and psychotic disorder (adjusted OR 0.58, 95% CI 0.36–0.93) with weaker evidence for moderate/severe depressive disorder (adjusted OR 0.86, 95% CI 0.73–1.01) and GAD (adjusted OR 0.89, 95% CI 0.78–1.02).

**Table 3:** Cross-sectional associations between plasma polyunsaturated fatty acid measures and odds of mental disorders at 24 years.

Outcome (*n* with vs. without outcome)	Exposure	Crude models			Adjusted models		
		Odds ratio	95% CI	*p*	Odds ratio	95% CI	*p*
Psychotic disorder (47 vs. 3842)	Total n-6 PUFAs	1.15	0.82–1.62	0.420	1.08	0.76–1.55	0.661
	Total n-3 PUFAs	0.66	0.41–1.06	0.083	0.71	0.45–1.12	0.141
	n-6:n-3 ratio	1.72	1.36–2.17	<0.001	1.54	1.16–2.03	0.003
	DHA % total fatty acids	0.55	0.36–0.85	0.007	0.58	0.36–0.93	0.025
Moderate/severe depressive disorder (304 vs. 3662)	Total n-6 PUFAs	1.07	0.93–1.23	0.366	0.99	0.85–1.15	0.898
	Total n-3 PUFAs	0.83	0.71–0.98	0.026	0.85	0.73–1.00	0.050
	n-6:n-3 ratio	1.37	1.20–1.56	<0.001	1.22	1.06–1.41	0.005
	DHA % total fatty acids	0.85	0.73–0.98	0.027	0.86	0.73–1.01	0.071
Generalised anxiety disorder (386 vs. 3571)	Total n-6 PUFAs	1.03	0.91–1.16	0.692	0.96	0.85–1.09	0.559
	Total n-3 PUFAs	0.88	0.77–1.00	0.053	0.88	0.77––1.01	0.064
	n-6:n-3 ratio	1.27	1.13–1.42	<0.001	1.17	1.03–1.32	0.017
	DHA % total fatty acids	0.90	0.79–1.02	0.085	0.89	0.78–1.02	0.094

*BMI* body mass index, *CI* confidence interval, *DHA* docosahexaenoic acid, *n-6* omega-6, *n-3* omega-3, *PUFA* polyunsaturated fatty acid.

PUFA measures were converted to z-scores such that the odds ratios may be interpreted as per standard deviation increase in the PUFA measure at 24 years. Covariates in adjusted models included precise age at attendance at age 24 clinic, sex, BMI at 24 years and average number of cigarettes smoked per day at 24 years.

#### Longitudinal associations between PUFAs at 17 years and incident mental disorders at 24 years

Results from logistic regression analyses examining associations between PUFA measures at 17 years and incident mental disorders at 24 years are presented in Table 4 and Fig. 1C. There was little evidence of associations between total n-6 fatty acids, total n-3 fatty acids or the n-6:n-3 ratio at age 17 and incident mental disorders at age 24. There was evidence of an inverse association between DHA % total fatty acids at age 17 and psychotic disorder at age 24 (adjusted OR 0.44, 95% CI 0.22–0.87) but comparatively little evidence for depressive disorder (adjusted OR 0.89, 95% CI 0.70–1.13) or GAD (adjusted OR 0.89, 95% CI 0.74–1.06).

**Table 4:** Longitudinal associations between plasma polyunsaturated fatty acid measures at 17 years and odds of incident mental disorders at 24 years.

Outcome (*n* with vs. without outcome)	Exposure	Crude models			Adjusted models		
		Odds ratio	95% CI	*p*	Odds ratio	95% CI	*p*
Psychotic disorder (20 vs. 2754)	Total n-6 PUFAs	1.14	0.71–1.82	0.590	1.06	0.63–1.78	0.824
	Total n-3 PUFAs	0.69	0.36–1.31	0.254	0.65	0.34–1.25	0.196
	n-6:n-3 ratio	1.43	0.94–2.16	0.092	1.42	0.93–2.16	0.105
	DHA % total fatty acids	0.44	0.23–0.86	0.016	0.44	0.22–0.87	0.019
Moderate/severe depressive disorder (157 vs. 2505)	Total n-6 PUFAs	1.11	0.90–1.36	0.332	0.98	0.78–1.24	0.876
	Total n-3 PUFAs	1.04	0.84–1.30	0.705	0.98	0.78–1.23	0.876
	n-6:n-3 ratio	1.05	0.81–1.35	0.703	1.02	0.79–1.32	0.852
	DHA % total fatty acids	0.94	0.75–1.18	0.595	0.89	0.70–1.13	0.334
Generalised anxiety disorder (205 vs. 2423)	Total n-6 PUFAs	1.26	1.07–1.48	0.006	1.14	0.95–1.37	0.156
	Total n-3 PUFAs	1.04	0.87–1.24	0.672	0.98	0.81–1.18	0.815
	n-6:n-3 ratio	1.17	0.97–1.41	0.096	1.14	0.95–1.37	0.169
	DHA % total fatty acids	0.91	0.77–1.09	0.311	0.89	0.74–1.06	0.193

*BMI* body mass index, *CI* confidence interval, *DHA* docosahexaenoic acid, *n-6* omega-6, *n-3* omega-3, *PUFA* polyunsaturated fatty acid.

PUFA measures were converted to z-scores such that the odds ratios may be interpreted as per standard deviation increase in the PUFA measure at 17 years. Covariates in adjusted models included age at attendance at age 17 clinic, sex, BMI at 17 years and average number of cigarettes smoked per day at 17 years.

#### Longitudinal associations between change in PUFAs from 17 to 24 years and incident mental disorders at 24 years

Results from logistic regression analyses examining associations between change in PUFA measures from 17 to 24 years and incident mental disorders at 24 years are presented in Supplementary Table 6. There was little evidence of associations between change in PUFA measures and incident mental disorders.

### Sensitivity analyses

There was little evidence of substantial confounding for the candidate variables examined based on change in effect estimates compared to the base models (Supplementary Tables 7–10).

## Discussion

We examined associations between plasma PUFAs and psychotic disorder, moderate/severe depressive disorder and GAD in adolescence and early adulthood in a general population sample. In general, there was little evidence of cross-sectional or longitudinal associations between total n-6 or total n-3 plasma PUFAs and mental disorders. There was also little evidence of cross-sectional associations between any PUFA measure and mental disorders at age 17. Cross-sectionally at age 24, the n-6:n-3 fatty acid ratio was positively associated with psychotic disorder, depressive disorder and GAD. There was evidence for an inverse cross-sectional association between DHA % total fatty acids and psychotic disorder at age 24, but little evidence for depression or GAD. In longitudinal analyses, total n-6 fatty acids, total n-3 fatty acids and the n-6:n-3 ratio at 17 years were not associated with incident mental disorders at 24 years. However, DHA % total fatty acids at age 17 was inversely associated with odds of incident psychotic disorder (but not depression or GAD) at age 24. Finally, there was little evidence of associations between change in PUFA measures from 17 to 24 years and incident mental disorders at 24 years.

Total n-6 and total n-3 levels were not associated with mental disorders cross-sectionally or longitudinally. This contrasts with evidence for cross-sectional associations between the ratio of n-6:n-3 fatty acids and mental disorders at age 24. This might suggest that it is the balance of n-6 to n-3 fatty acids that is of greater importance in relation to these disorders in early adulthood rather than their absolute levels. One possible explanation is that a higher n-6:n-3 ratio predisposes to increased inflammation^4^. Higher levels of circulating inflammatory biomarkers have been detected in association with and preceding the onset of several mental disorders compared to controls^46–50^. Increasing the proportion of n-3 PUFAs while decreasing n-6 PUFAs could potentially reduce risk of mental illness (at least in part) by reduction of neuroinflammation, for example via increased synthesis of specialised pro-resolving mediators by n-3 fatty acids^51^. However, it remains unclear whether supplementation with n-3 PUFAs, either alone or in combination with dietary changes to reduce the n-6:n-3 ratio, is sufficient to induce putative protective effects on risk of mental illness as mediated via these inflammatory mechanisms^52,53^.

We observed cross-sectional associations between DHA and psychotic disorder at age 24, but not age 17. These findings could suggest that adult-onset psychotic disorder is more associated with PUFA abnormalities than early-onset psychosis. Early-onset psychosis may differ from adult-onset illness in terms of genetic liability and neurodevelopmental trajectory^54,55^, but whether or how these differences relate to PUFA status is unclear and will require further evaluation in future studies. Longitudinally, higher levels of DHA at age 17 were associated with lower odds of psychotic disorder at age 24. Given the low incidence of psychotic disorder in the longitudinal sample (0.7% of participants who were assessed at both timepoints) these results should be interpreted with caution. We can however speculate that any beneficial effects of DHA on reducing psychosis risk are dependent on developmental stage. Indeed, there was little evidence that changes in PUFAs from 17 to 24 years were associated with incident psychosis (Supplementary Table 6). This could indicate that adolescent PUFA status may be of greater importance in relation to psychosis risk in early adulthood than change in PUFA status after adolescence. Such sensitivity to developmental stage could also be one reason why clinical trials of n-3 fatty acids in schizophrenia generally suggest greater efficacy in reducing psychotic symptoms in early stages of the disorder compared to patients with chronic illness^56^ and is also in keeping with the typical age of onset of schizophrenia in early adulthood^57^.

Aside from modulating neuroinflammatory processes as discussed above, DHA has further neurobiological effects that may be of particular relevance to psychosis. The process of synaptic pruning extends normatively through adolescence and early adulthood^58^. Complement system proteins tag synapses for elimination by microglia^59^. Microglial activation, leading to excessive synaptic pruning, is thought to contribute to the pathogenesis of schizophrenia^60^. DHA is known to modulate activity of glial cells^61^. It could be speculated that higher levels of DHA may ‘buffer’ against excessive synaptic pruning through modulation of complement system and microglial activity^14,62^. DHA is also thought to play a role in dopaminergic neural development and survival^63^. It has been hypothesised that n-3 PUFA deficiency during critical phases of brain development may give rise to dopaminergic dysfunction as observed in schizophrenia^63^. It is also possible that DHA depletion occurs as a consequence of underlying biological abnormalities that have been observed in association with schizophrenia, including genetic variation in fatty acid desaturase enzymes, abnormalities in fatty acid binding proteins, pathologically enhanced phospholipase A2 activity, increased oxidative stress and gut microbial dysbiosis (see^64^ for a review of these factors).

It should be acknowledged that given the observational nature of this study, it is not possible to ascribe causality, especially for the cross-sectional analyses. It may be the case, for example, that participants with mental disorders in early adulthood ate a poorer diet (lower in n-3 PUFAs or with a higher ratio of n-6 to n-3 fatty acids) as a result of the functional impact of their symptoms. Nonetheless, the same associations were not observed cross-sectionally at age 17 years using the same outcome definitions, which together with the longitudinal findings potentially suggests that reverse causality is not the sole explanation, at least in the case of psychotic disorder. On the other hand, in cross-sectional analyses at age 17 years, participants may have been more likely to follow the diet of their family or carers rather than their own individual dietary choices (which might emerge to a greater extent in early adulthood).

It is unlikely that PUFA status measured at a single point in time is sufficient to predict risk of future psychotic illness, but rather that longer-term deficiency in DHA, particularly in early development and adolescence, leads to enhanced risk in concert with other genetic and environmental factors. In interpreting our findings, we make an underlying presumption that the ‘snapshot’ provided by plasma levels at least partially reflects PUFA status over a longer duration of exposure. There is evidence that overall adolescent dietary patterns remain moderately stable over time^65–69^ but, without access to shorter-interval repeated measures of plasma PUFAs and dietary information, we cannot definitively measure the accuracy of this presumption in the present data.

Previous general population studies have reported on associations between PUFAs and psychosis phenotypes. For example, a longitudinal study of Swedish women found that those who ate fish 3–4 times per week had reduced risk of subsequent high-frequency psychotic experiences compared to women who never ate fish^70^. However, previous investigations in the ALSPAC cohort have found limited evidence of associations between plasma PUFA measures in childhood or mid-adolescence in relation to psychotic experiences or disorder in late adolescence^71,72^. Studies have reported inverse associations between dietary PUFA intake and depression^73,74^ and anxiety disorders^75,76^ in the general population, although conflicting results have also been reported in studies of samples of predominantly older adults^77,78^. A study in adolescents reported an inverse association between self-reported n-3 PUFA intake at age 14 years and depressive symptoms at age 17 years, although this did not persist when adjusted for confounding factors^79^. In a longitudinal study of young adults at clinical high risk for psychosis, higher baseline n-6:n-3 ratio and lower DHA levels were predictive of onset of mood disorder on follow-up, but not anxiety or psychosis^80^. Mixed results have been reported in trials of PUFAs as treatments for mental disorders, although meta-analyses suggest that EPA (rather than DHA) supplementation may have some efficacy in treatment of depression^81^ and that high-dose n-3 preparations may reduce anxiety symptoms^82^. It may be the case that only a subset of patients with already-identifiable PUFA abnormalities benefit optimally from these interventions^64^.

Regarding implications for prevention, the present data provide tentative evidence to support a role for DHA in adolescence in relation to prevention of psychosis in early adulthood. The results provide less support for prevention of depression or anxiety, in keeping with a systematic review of clinical trials which found that n-3 PUFA supplementation had little effect in prevention of depressive or anxiety symptoms^83^. Independent validation of the current findings in samples with larger incidence of psychosis (such as in clinical high-risk for psychosis populations)^28^ is required. While we examined multiple potential confounders, the possibility of residual confounding cannot be excluded. Studies implementing causal methods such as Mendelian randomisation may be helpful in determining whether PUFAs (and particularly DHA) have a causal role in development of psychosis. Further studies examining PUFA exposure earlier in development, such as in early childhood, may be helpful in evaluating longer-term associations with risk of mental disorders in later life. Considering our findings of a longitudinal inverse association between DHA in adolescence and odds of incident psychotic disorder, large-scale adequately powered trials of DHA supplementation or dietary intervention in adolescence with long-term follow-up may facilitate investigation of DHA as a modifiable preventative factor in relation to risk of psychosis and other mental disorders in early adulthood.

### Strengths and limitations

Strengths of this study include its focus on a well-characterised large general population cohort of young adults; the use of biomarkers of PUFA status rather than self-reported intake; and the availability of repeated measures enabling longitudinal analyses. However, several limitations should be noted. In common with most longitudinal studies, the ALSPAC sample is subject to attrition. This occurred along a socio-economic gradient and may limit generalisability. Exposure and confounder variables had varying amounts of missing data, and participants differed on several characteristics depending on whether PUFA data were available. We attempted to address this using multiple imputation. Unlike the CIS-R, the PLIKSi does not provide ICD-10-defined diagnoses. However, individuals who fulfilled the definition of psychotic disorder in our study would also likely meet ICD criteria since they were experiencing regular psychotic symptoms associated with severe distress or impairment. The low number of incident cases of psychosis in the longitudinal sample (*n* = 20) may increase the chance of type I error. We did not have access to data for other PUFAs (for example EPA and nervonic acid) and were unable to assess their influence. It is possible that unmeasured PUFAs have stronger or weaker associations with certain mental disorders that cannot be evaluated in this study^14,81^. Unobserved dietary components (such as fruit and vegetable intake or supplement use) and non-dietary factors (such as physical activity) may residually confound the observed associations. While nuclear magnetic resonance spectroscopy facilitates high-throughput reproducible analyses, other methods (such as mass spectrometry) may have higher sensitivity^84^ and it is possible that the observed associations may thus be under-estimated due to measurement error. Samples were stored for variable ranges of time prior to analysis during which degradation could have occurred. There is evidence that PUFAs are reasonably stable for up to seven years of storage^85,86^, however without access to individual-level data for storage time we were unable to investigate this. Finally, in contrast to much of the existing literature, plasma PUFA levels were measured as opposed to erythrocyte membrane PUFA composition. While plasma measures are considered robust biomarkers of PUFA status^87^, there are two potential associated limitations. Firstly, plasma levels may correlate less strongly with brain PUFA content in comparison to erythrocyte membrane levels, although this is uncertain in humans^88,89^. Secondly, plasma levels reflect PUFA status over a shorter timeframe (~1–2 weeks) than membrane composition (~1–2 months) because the latter has slower turnover^90^. Thus, membrane levels would likely provide a more accurate reflection of long-term PUFA status.

## Conclusions

We report evidence of cross-sectional associations between the n-6:n-3 fatty acid ratio and mental disorders in early adulthood, but not late adolescence, in a general population sample. There was evidence of inverse associations between DHA and odds of psychotic disorder cross-sectionally in early adulthood and longitudinally in late adolescence. Long-term trials specifically in adolescent and clinical high-risk for psychosis samples may further clarify the potential role of PUFAs in prevention of mental disorders.

## Supplementary information

Supplemental Material

## References

[1] Schmitz G, Ecker J (2008). The opposing effects of n−3 and n−6 fatty acids. Prog. Lipid Res..

[2] Burdge G (2004). Alpha-linolenic acid metabolism in men and women: nutritional and biological implications. Curr. Opin. Clin. Nutr. Metab. Care..

[3] Simopoulos AP (2006). Evolutionary aspects of diet, the omega-6/omega-3 ratio and genetic variation: nutritional implications for chronic diseases. Biomed. Pharmacother. (Biomedecine pharmacotherapie).

[4] DiNicolantonio JJ, O’Keefe JH (2018). Importance of maintaining a low omega-6/omega-3 ratio for reducing inflammation. Open Heart.

[5] Khandaker GM, Dantzer R, Jones PB (2017). Immunopsychiatry: important facts. Psychol. Med..

[6] Buckley PF (2019). Neuroinflammation and schizophrenia. Curr. Psychiatry Rep..

[7] Baumeister D, Russell A, Pariante CM, Mondelli V (2014). Inflammatory biomarker profiles of mental disorders and their relation to clinical, social and lifestyle factors. Soc. Psychiatry Psychiatr. Epidemiol..

[8] Mongan D, Ramesar M, Föcking M, Cannon M, Cotter D (2020). Role of inflammation in the pathogenesis of schizophrenia: a review of the evidence, proposed mechanisms and implications for treatment. Early Intervention Psychiatry.

[9] Reimers A, Ljung H (2019). The emerging role of omega-3 fatty acids as a therapeutic option in neuropsychiatric disorders. Ther. Adv. Psychopharmacol..

[10] Turk HF, Chapkin RS (2013). Membrane lipid raft organization is uniquely modified by n-3 polyunsaturated fatty acids. Prostaglandins Leukot. Essent Fatty Acids.

[11] Cutuli D (2017). Functional and structural benefits induced by omega-3 polyunsaturated fatty acids during aging. Curr. Neuropharmacol..

[12] Weiser MJ, Butt CM, Mohajeri MH (2016). Docosahexaenoic acid and cognition throughout the lifespan. Nutrients.

[13] Che H (2018). Neuroprotective effects of n-3 polyunsaturated fatty acid-enriched phosphatidylserine against oxidative damage in PC12 Cells. Cell Mol. Neurobiol..

[14] Dyall SC (2015). Long-chain omega-3 fatty acids and the brain: a review of the independent and shared effects of EPA, DPA and DHA. Front Aging Neurosci..

[15] Hoen WP (2013). Red blood cell polyunsaturated fatty acids measured in red blood cells and schizophrenia: a meta-analysis. Psychiatry Res..

[16] Lin PY, Huang SY, Su KP (2010). A meta-analytic review of polyunsaturated fatty acid compositions in patients with depression. Biol. Psychiatry.

[17] Ross BM (2009). Omega-3 polyunsaturated fatty acids and anxiety disorders. Prostaglandins, Leukotrienes Essent. Fat. Acids.

[18] Chang JP-C (2019). High-dose eicosapentaenoic acid (EPA) improves attention and vigilance in children and adolescents with attention deficit hyperactivity disorder (ADHD) and low endogenous EPA levels. Trans. Psychiatry.

[19] Mazahery H (2017). Relationship between long chain n-3 polyunsaturated fatty acids and autism spectrum disorder: systematic review and meta-analysis of Case-Control and Randomised Controlled Trials. Nutrients.

[20] Cole GM, Ma Q-L, Frautschy SA (2009). Omega-3 fatty acids and dementia. Prostaglandins Leukot. Ess. Fat. Acids.

[21] Saraceno B, Levav I, Kohn R (2005). The public mental health significance of research on socio-economic factors in schizophrenia and major depression. World Psychiatry.: Off. J. World Psychiatr. Assoc..

[22] Livingstone K. M. et al. Socioeconomic inequities in diet quality and nutrient intakes among Australian adults: findings from a nationally representative cross-sectional study. *Nutrients*. **9**, 1092 (2017). 10.3390/nu9101092 PMC5691709 28976927

[23] Darmon N, Drewnowski A (2008). Does social class predict diet quality?. Am. J. Clin. Nutr..

[24] Firth J (2019). The efficacy and safety of nutrient supplements in the treatment of mental disorders: a meta-review of meta-analyses of randomized controlled trials. World Psychiatry.: Off. J. World Psychiatr. Assoc..

[25] Amminger GP (2010). Long-chain omega-3 fatty acids for indicated prevention of psychotic disorders: a randomized, placebo-controlled trial. Arch. Gen. Psychiatry.

[26] McGorry PD (2017). Effect of ω-3 polyunsaturated fatty acids in young people at ultrahigh risk for psychotic disorders: the NEURAPRO randomized clinical trial. JAMA Psychiatry.

[27] Berger M. et al. Relationship between polyunsaturated fatty acids and psychopathology in the NEURAPRO Clinical Trial. *Front. Psychiatry*. **10**, 393 (2019). 10.3389/fpsyt.2019.00393 PMC6562242 31244693

[28] Amminger GP (2020). The NEURAPRO biomarker analysis: long-chain omega-3 fatty acids improve 6-month and 12-month outcomes in youths at ultra-high risk for psychosis. Biol. Psychiatry.

[29] de Girolamo G, Dagani J, Purcell R, Cocchi A, McGorry PD (2012). Age of onset of mental disorders and use of mental health services: needs, opportunities and obstacles. Epidemiol. Psychiatr. Sci..

[30] Boyd A (2013). Cohort Profile: the ‘children of the 90s’-the index offspring of the Avon Longitudinal Study of Parents and Children. Int J. Epidemiol..

[31] Fraser A (2013). Cohort Profile: the avon longitudinal study of parents and children: ALSPAC mothers cohort. Int J. Epidemiol..

[32] Northstone K. et al. The Avon Longitudinal Study of Parents and Children (ALSPAC): an update on the enrolled sample of index children in 2019 [version 1; peer review: 2 approved]. Wellcome Open Research. 2019;4. 10.12688/wellcomeopenres.15132.1 PMC6464058 31020050

[33] Harris PA (2009). Research electronic data capture (REDCap)—A metadata-driven methodology and workflow process for providing translational research informatics support. J. Biomed. Inform..

[34] Harris PA (2019). The REDCap consortium: building an international community of software platform partners. J. Biomed. Inform..

[35] Soininen P (2009). High-throughput serum NMR metabonomics for cost-effective holistic studies on systemic metabolism. Analyst.

[36] Horwood J (2008). IQ and non-clinical psychotic symptoms in 12-year-olds: results from the ALSPAC birth cohort. The. Br. J. Psychiatry.: J. Ment. Sci..

[37] World Health Organisation. Division of Mental Health. Schedules for clinical assessment in neuropsychiatry: version 2. World Health Organisation; 1994.

[38] Zammit S (2013). Psychotic experiences and psychotic disorders at Age 18 in relation to psychotic experiences at age 12 in a longitudinal population-based cohort study. Am. J. Psychiatry.

[39] Sullivan SA (2020). A population-based cohort study examining the incidence and impact of psychotic experiences from childhood to adulthood, and prediction of psychotic disorder. Am. J. Psychiatry.

[40] Lewis G (1994). Assessing psychiatric disorder with a human interviewer or a computer. J. Epidemiol. Community Health.

[41] World Health Organisation. (2004). ICD-10: international statistical classification of diseases and related health problems: tenth revision..

[42] de Groot RHM, Emmett R, Meyer BJ (2019). Non-dietary factors associated with n-3 long-chain PUFA levels in humans - a systematic literature review. Br. J. Nutr..

[43] World Health Organisation. AUDIT: the Alcohol Use Disorders Identification Test: guidelines for use in primary health care/Thomas F. Babor et al. 2nd ed. Geneva: World Health Organization (2001).

[44] Bush K, Kivlahan DR, McDonell MB, Fihn SD, Bradley KA (1998). The AUDIT alcohol consumption questions (AUDIT-C): an effective brief screening test for problem drinking. Ambulatory Care Quality Improvement Project (ACQUIP). Alcohol Use Disorders Identification Test. Arch. Intern. Med..

[45] Wechsler D. Wechsler Intelligence Scale for Children (3rd ed.). San Antonio, Texas: The Psychological Corporation; 1991.

[46] Khandaker GM, Pearson RM, Zammit S, Lewis G, Jones PB (2014). Association of serum interleukin 6 and C-reactive protein in childhood with depression and psychosis in young adult life: a population-based longitudinal study. JAMA Psychiatry.

[47] Miller BJ, Goldsmith DR (2019). Inflammatory biomarkers in schizophrenia: Implications for heterogeneity and neurobiology. Biomark. Neuropsychiatry.

[48] Costello H, Gould RL, Abrol E, Howard R (2019). Systematic review and meta-analysis of the association between peripheral inflammatory cytokines and generalised anxiety disorder. BMJ Open..

[49] Goldsmith DR, Rapaport MH, Miller BJ (2016). A meta-analysis of blood cytokine network alterations in psychiatric patients: comparisons between schizophrenia, bipolar disorder and depression. Mol. Psychiatry.

[50] Köhler CA (2017). Peripheral cytokine and chemokine alterations in depression: a meta-analysis of 82 studies. Acta Psychiatr. Scand..

[51] Joffre C, Rey C, Layé S (2019). N-3 polyunsaturated fatty acids and the resolution of neuroinflammation. Front. Pharmacol..

[52] Aucoin M, LaChance L, Cooley K, Kidd S (2020). Diet and psychosis: a scoping review. Neuropsychobiology.

[53] Marx W, Moseley G, Berk M, Jacka F (2017). Nutritional psychiatry: the present state of the evidence. Proc. Nutr. Soc..

[54] Fernandez A (2019). Childhood-onset schizophrenia: a systematic overview of its genetic heterogeneity from classical studies to the genomic era. Front Genet..

[55] Ahn K (2014). High rate of disease-related copy number variations in childhood onset schizophrenia. Mol. Psychiatry.

[56] Chen AT, Chibnall JT, Nasrallah HA (2015). A meta-analysis of placebo-controlled trials of omega-3 fatty acid augmentation in schizophrenia: possible stage-specific effects. Ann. Clin. Psychiatry.

[57] Gogtay N, Vyas NS, Testa R, Wood SJ, Pantelis C (2011). Age of onset of schizophrenia: perspectives from structural neuroimaging studies. Schizophr. Bull..

[58] Petanjek Z (2011). Extraordinary neoteny of synaptic spines in the human prefrontal cortex. Proc. Natl Acad. Sci. USA.

[59] Presumey J, Bialas AR, Carroll MC (2017). Complement system in neural synapse elimination in development and disease. Adv. Immunol..

[60] Sellgren CM (2019). Increased synapse elimination by microglia in schizophrenia patient-derived models of synaptic pruning. Nat. Neurosci..

[61] Heras-Sandoval D, Pedraza-Chaverri J, Pérez-Rojas JM (2016). Role of docosahexaenoic acid in the modulation of glial cells in Alzheimer’s disease. J. Neuroinflammation..

[62] Madore C (2020). Essential omega-3 fatty acids tune microglial phagocytosis of synaptic elements in the mouse developing brain. Nat. Commun..

[63] Healy-Stoffel M, Levant B (2018). N-3 (Omega-3) fatty acids: effects on brain dopamine systems and potential role in the etiology and treatment of neuropsychiatric disorders. CNS Neurol. Disord. Drug Targets.

[64] Hsu M-C, Huang Y-S, Ouyang W-C (2020). Beneficial effects of omega-3 fatty acid supplementation in schizophrenia: possible mechanisms. Lipids Health Dis..

[65] Cutler GJ, Flood A, Hannan P, Neumark-Sztainer D (2009). Major patterns of dietary intake in adolescents and their stability over time. J. Nutr..

[66] Movassagh EZ, Baxter-Jones ADG, Kontulainen S, Whiting SJ, Vatanparast H (2017). Tracking dietary patterns over 20 years from childhood through adolescence into young adulthood: the Saskatchewan pediatric bone mineral accrual study. Nutrients.

[67] Ambrosini GL, Emmett PM, Northstone K, Jebb SA (2014). Tracking a dietary pattern associated with increased adiposity in childhood and adolescence. Obesity.

[68] Biazzi Leal D (2017). Changes in dietary patterns from childhood to adolescence and associated body adiposity status. Nutrients.

[69] Harris C (2015). Changes in dietary intake during puberty and their determinants: results from the GINIplus birth cohort study. BMC Public Health.

[70] Hedelin M (2010). Dietary intake of fish, omega-3, omega-6 polyunsaturated fatty acids and vitamin D and the prevalence of psychotic-like symptoms in a cohort of 33 000 women from the general population. BMC Psychiatry.

[71] Ramsay H (2018). Cognition, psychosis risk and metabolic measures in two adolescent birth cohorts. Psychol. Med..

[72] Thompson AD (2020). Omega-3 and Omega-6 fatty acids and risk of psychotic outcomes in the ALSPAC birth cohort. Schizophr. Res..

[73] Lai JS (2014). A systematic review and meta-analysis of dietary patterns and depression in community-dwelling adults. Am. J. Clin. Nutr..

[74] Grosso G (2016). Dietary n-3 PUFA, fish consumption and depression: a systematic review and meta-analysis of observational studies. J. Affect Disord..

[75] Jacka FN (2013). Dietary intake of fish and PUFA, and clinical depressive and anxiety disorders in women. Br. J. Nutr..

[76] Natacci L (2018). Omega 3 consumption and anxiety disorders: a cross-sectional analysis of the Brazilian Longitudinal Study of Adult Health (ELSA-Brasil). Nutrients.

[77] Astorg P (2009). Long-chain n-3 fatty acid levels in baseline serum phospholipids do not predict later occurrence of depressive episodes: a nested case-control study within a cohort of middle-aged French men and women. Prostaglandins Leukot. Ess. Fat. Acids.

[78] Persons JE (2014). Omega-3 fatty acid biomarkers and subsequent depressive symptoms. Int J. Geriatr. Psychiatry.

[79] Oddy WH (2011). Dietary intake of omega-3 fatty acids and risk of depressive symptoms in adolescents. Depression Anxiety.

[80] Berger ME (2017). Omega-6 to omega-3 polyunsaturated fatty acid ratio and subsequent mood disorders in young people with at-risk mental states: a 7-year longitudinal study. Transl. Psychiatry.

[81] Liao Y (2019). Efficacy of omega-3 PUFAs in depression: a meta-analysis. Transl. Psychiatry.

[82] Su K-P (2018). Association of use of omega-3 polyunsaturated fatty acids with changes in severity of anxiety symptoms: a systematic review and meta-analysis. JAMA Netw. Open..

[83] Deane KHO (2021). Omega-3 and polyunsaturated fat for prevention of depression and anxiety symptoms: systematic review and meta-analysis of randomised trials. Br. J. Psychiatry.

[84] Emwas A-H, Salek R, Griffin J, Merzaban J (2013). NMR-based metabolomics in human disease diagnosis: applications, limitations, and recommendations. Metabolomics.

[85] Jonasdottir HS, Brouwers H, Toes REM, Ioan-Facsinay A, Giera M (2018). Effects of anticoagulants and storage conditions on clinical oxylipid levels in human plasma. Biochim. Biophys. Acta Mol. Cell Biol. Lipids.

[86] Wagner-Golbs A. et al. Effects of long-term storage at -80 °C on the human plasma metabolome. *Metabolites*. **9**, 99 (2019). 10.3390/metabo9050099 PMC6572224 31108909

[87] Serra-Majem L, Nissensohn M, Øverby NC, Fekete K (2012). Dietary methods and biomarkers of omega 3 fatty acids: a systematic review. Br. J. Nutr..

[88] Guest J, Garg M, Bilgin A, Grant R (2013). Relationship between central and peripheral fatty acids in humans. Lipids Health Dis..

[89] Kuratko CN, Salem N (2009). Biomarkers of DHA status. Prostaglandins Leukotrienes Essent. Fat. Acids.

[90] Sun Q, Ma J, Campos H, Hankinson SE, Hu FB (2007). Comparison between plasma and erythrocyte fatty acid content as biomarkers of fatty acid intake in US women. Am. J. Clin. Nutr..

