# Supplementary material for: Plasma polyunsaturated fatty acids and mental disorders in adolescence and early adulthood: cross-sectional and longitudinal associations in a general population cohort
Source: Transl Psychiatry. 2021 May 31;11:321. doi: 10.1038/s41398-021-01425-4 (PMC8167090; doi:10.1038/s41398-021-01425-4)
Supplement: Supplementary file 1 — Supplemental Material [file 41398_2021_1425_MOESM1_ESM.docx]

**Supplementary Information**

| Supplementary Methods | 2 |
| --- | --- |
| Supplementary References | 5 |
| Supplementary Table 1: Comparison of sociodemographic characteristics for participants who did versus did not attend age 17 and/or age 24 clinics | 7 |
| Supplementary Table 2: Comparison of characteristics for participants with and without polyunsaturated fatty acid data available at age 17 years (total age 17 sample *n*=5215) | 8 |
| Supplementary Table 3: Comparison of characteristics for participants with and without polyunsaturated fatty acid data available at age 24 years (total age 24 sample *n*=4019) | 10 |
| Supplementary Table 4: Characteristics of age 17 sample according to specific mental disorder at age 17 years | 12 |
| Supplementary Table 5: Characteristics of age 24 sample according to specific mental disorder at age 24 years | 15 |
| Supplementary Table 6: Effect estimates for associations between change in plasma polyunsaturated fatty acid measures from 17 to 24 years and odds of incident mental disorders at 24 years | 18 |
| Supplementary Table 7: Odds ratios for cross-sectional associations between PUFA measures and mental disorders at age 17 years in models with additional adjustment for further potential confounding factors | 19 |
| Supplementary Table 8: Odds ratios for cross-sectional associations between PUFA measures and mental disorders at age 24 years in models with additional adjustment for further potential confounding factors | 20 |
| Supplementary Table 9: Odds ratios for longitudinal associations between PUFA measures at age 17 years and incident mental disorders at age 24 years in models with additional adjustment for further potential confounding factors | 21 |
| Supplementary Table 10: Odds ratios for longitudinal associations between change in PUFA measures from age 17 to age 24 years and incident mental disorders at age 24 years in models with additional adjustment for further potential confounding factors | 22 |
| Supplementary Figure 1: Participant flow diagram depicting sample derivation | 23 |

**Supplementary Methods**

**Further details regarding confounder variables**

*Age, sex, BMI, tobacco smoking*

Age, sex, BMI and tobacco smoking were included as confounders on the basis of a recent systematic review of non-dietary factors associated with n-3 PUFA levels in humans (1). At both age 17 and age 24 clinics, weight was measured using electronic scales to the nearest 50g and height with a Harpenden stadiometer to the nearest cm. The tobacco smoking variable was the average number of cigarettes smoked per day in the preceding 30 days, as derived from a questionnaire regarding tobacco smoking history administered at both age 17 and age 24 clinics.

*Alcohol intake*

There is little evidence for a general association between alcohol consumption and polyunsaturated fatty acids (PUFAs), although there is evidence for an association with wine drinking specifically (1). It has therefore been recommended to consider adjusting for alcohol, but specified according to type and amount (1). We did not have access to data on the specific types of alcohol consumed. However, at age 17 participants completed the Alcohol Use Disorders Identification Test (AUDIT) (2). The AUDIT contains questions on alcohol consumption, dependence behaviour and alcohol-related problems with a maximum score of 40 points. At age 24 participants completed the Alcohol Use Disorders Identification Test – Concise (AUDIT-C) (3), a shorter screening form of the AUDIT, with a maximum score of 12 points.

*Regular cannabis use*

PUFAs may interact biologically with endocannabinoid metabolism (4, 5). Cannabis use may be associated with psychotic, depressive and anxiety symptoms (6-10). We hence examined regular use of cannabis as a potential confounding factor.

At age 17, participants were asked about frequency of cannabis use in the past year: monthly or less; 2 – 4 times per month; 2 – 3 times per week; or 4+ times per week. We collapsed the latter three categories and defined regular cannabis use at age 17 years as using cannabis more than once monthly (yes/ no).

At age 24, participants were again asked about frequency of cannabis use in the past year: not in the past year; once or twice; less than monthly; weekly; daily or almost daily. We collapsed the latter two categories and defined regular cannabis use at age 24 years as using cannabis on a weekly or daily basis (yes/ no).

*Prenatal and childhood socio-economic factors*

Given evidence of associations between socio-economic status and PUFA intake (11, 12) and between socio-economic status and mental disorders (13) we considered several socio-economic variables as potential confounders.

We examined family home ownership status in early pregnancy (own home or mortgage/ rent or other) as measured by questionnaire completed by mothers at 8 weeks gestation. We examined social class (manual/ non-manual) of the participant’s mother or father (whichever was highest) as measured by a questionnaire completed by mothers at 32 weeks gestation. We also considered highest maternal educational qualification as measured by questionnaire at 32 weeks gestation (less than O-level/ O-level or higher). O-levels are the standard educational qualification taken by UK students at approximately age 16 years. With respect to childhood, we examined average take-home family income as measured by a questionnaire completed by mothers when the participant was aged approximately 8 years (<£400 per week/ £400 or more per week).

*Childhood IQ*

PUFA measures may be associated with cognitive function (14). Higher IQ in childhood may be associated with healthier dietary choices in later life (15) while lower IQ may be associated with increased risk of mental disorders (16, 17). ALSPAC participants were invited to attend a clinic when aged approximately 8 years old at which IQ was measured using the Wechsler Intelligence Scale for Children (Third Edition) (18). We examined the total IQ score derived from this measure as a potential confounder.

**Further details regarding multiple imputation**

We used multiple imputation with chained equations to impute missing data for exposures and covariates. In addition to the outcomes, exposures and confounders included above and in the main analyses, we included several auxiliary variables in the imputation model (based on their relationship with the outcomes of interest and/or missingness) to make the missing-at-random assumption more plausible. These included: the participant’s ethnicity (white/ non-white); BMI at age 11; psychotic experiences at age 12 according to the Psychosis-Like Symptom Interview (none/ suspected/ definite); Short Mood and Feelings Questionnaire (19) score at age 12; whether or not the participant had smoked tobacco at age 12; whether or not the participant had drank alcohol at age 12; whether or not the participant had tried cannabis by age 12; whether or not the participant was in education or employment at age 17; Cognitive Style Questionnaire – Short Form (20) score at age 17; whether or not the participant reported having thoughts of self-harm in the past week at age 17; whether or not the participant had experienced trauma (physical abuse, emotional abuse, domestic violence, sexual abuse or bullying) from birth to age 17 (see (21) for further details); whether or not the participant had reported being in trouble with police in the past year at age 17; whether or not the participant was in employment, education or training at age 24; whether or not the participant reported a history of previous self-harm at age 24; the father’s highest educational qualification prior to birth of the child; whether or not the pregnancy was intentional; whether or not the mother smoked tobacco during the first trimester of pregnancy; maternal Edinburgh Postnatal Depression Scale (22) score measured at 18 weeks gestation; maternal birth age; and the Family Adversity Index (FAI) score (23). The FAI is a cumulative measure consisting of 18 items assessed by postal questionnaires completed by mothers of ALSPAC participants during pregnancy. The specific items included related to multiple socio-economic factors including maternal age and education, housing, financial difficulties, partner relationship, family size, social support, maternal affective disorder, substance abuse and crime. Each individual adversity item was rated one if present and zero if not present, then summed to derive a total score.

Binary variables were imputed in the imputation model using logistic regression, categorical variables using multinomial regression and ordinal variables using ordinal regression. Continuous variables were imputed using predictive mean matching with *k*=10 nearest neighbours. Fifty imputed datasets were created and estimates were combined according to Rubin’s rules (24).

**Supplementary References**

1. de Groot RHM, Emmett R, Meyer BJ. Non-dietary factors associated with n-3 long-chain PUFA levels in humans - a systematic literature review. Br J Nutr. 2019;121(7):793-808.

2. World Health Organisation. AUDIT: the Alcohol Use Disorders Identification Test : guidelines for use in primary health care / Thomas F. Babor ... [‎‎et al.]‎‎. 2nd ed ed. Geneva: World Health Organization; 2001.

3. Bush K, Kivlahan DR, McDonell MB, Fihn SD, Bradley KA. The AUDIT alcohol consumption questions (AUDIT-C): an effective brief screening test for problem drinking. Ambulatory Care Quality Improvement Project (ACQUIP). Alcohol Use Disorders Identification Test. Archives of internal medicine. 1998;158(16):1789-95.

4. Watson JE, Kim JS, Das A. Emerging class of omega-3 fatty acid endocannabinoids & their derivatives. Prostaglandins Other Lipid Mediat. 2019;143:106337-.

5. Dyall SC. Interplay Between n-3 and n-6 Long-Chain Polyunsaturated Fatty Acids and the Endocannabinoid System in Brain Protection and Repair. Lipids. 2017;52(11):885-900.

6. Shakoor S, Zavos HMS, McGuire P, Cardno AG, Freeman D, Ronald A. Psychotic experiences are linked to cannabis use in adolescents in the community because of common underlying environmental risk factors. Psychiatry research. 2015;227(2-3):144-51.

7. Fergusson DM, Horwood LJ, Swain-Campbell NR. Cannabis dependence and psychotic symptoms in young people. Psychological medicine. 2003;33(1):15-21.

8. Lev-Ran S, Roerecke M, Le Foll B, George TP, McKenzie K, Rehm J. The association between cannabis use and depression: a systematic review and meta-analysis of longitudinal studies. Psychological medicine. 2014;44(4):797-810.

9. Hayatbakhsh MR, Najman JM, Jamrozik K, Mamun AA, Alati R, Bor W. Cannabis and anxiety and depression in young adults: a large prospective study. Journal of the American Academy of Child and Adolescent Psychiatry. 2007;46(3):408-17.

10. Horwood LJ, Fergusson DM, Coffey C, Patton GC, Tait R, Smart D, et al. Cannabis and depression: An integrative data analysis of four Australasian cohorts. Drug and Alcohol Dependence. 2012;126(3):369-78.

11. Livingstone KM, Olstad DL, Leech RM, Ball K, Meertens B, Potter J, et al. Socioeconomic Inequities in Diet Quality and Nutrient Intakes among Australian Adults: Findings from a Nationally Representative Cross-Sectional Study. Nutrients. 2017;9(10):1092.

12. Darmon N, Drewnowski A. Does social class predict diet quality? The American journal of clinical nutrition. 2008;87(5):1107-17.

13. Saraceno B, Levav I, Kohn R. The public mental health significance of research on socio-economic factors in schizophrenia and major depression. World psychiatry : official journal of the World Psychiatric Association (WPA). 2005;4(3):181-5.

14. Ramsay H, Barnett JH, Murray GK, Miettunen J, Mäki P, Järvelin MR, et al. Cognition, psychosis risk and metabolic measures in two adolescent birth cohorts. Psychological medicine. 2018;48(15):2609-23.

15. Wraw C, Der G, Gale CR, Deary IJ. Intelligence in youth and health behaviours in middle age. Intelligence. 2018;69:71-86.

16. Zammit S, Allebeck P, David AS, Dalman C, Hemmingsson T, Lundberg I, et al. A longitudinal study of premorbid IQ Score and risk of developing schizophrenia, bipolar disorder, severe depression, and other nonaffective psychoses. Archives of general psychiatry. 2004;61(4):354-60.

17. Koenen KC, Moffitt TE, Roberts AL, Martin LT, Kubzansky L, Harrington H, et al. Childhood IQ and adult mental disorders: a test of the cognitive reserve hypothesis. The American journal of psychiatry. 2009;166(1):50-7.

18. Wechsler D. Wechsler Intelligence Scale for Children (3rd ed.). San Antonio, Texas: The Psychological Corporation; 1991.

19. Ancold A, Stephen C. Development of a short questionnaire for use in epidemiological studies of depression in children and adolescents. Age (years). 1995;6(11).

20. Meins E, McCarthy-Jones S, Fernyhough C, Lewis G, Bentall RP, Alloy LB. Assessing negative cognitive style: Development and validation of a Short-Form version of the Cognitive Style Questionnaire. Personality and individual differences. 2012;52(5):581-5.

21. Croft J, Heron J, Teufel C, Cannon M, Wolke D, Thompson A, et al. Association of Trauma Type, Age of Exposure, and Frequency in Childhood and Adolescence With Psychotic Experiences in Early Adulthood. JAMA psychiatry. 2018.

22. Cox JL, Holden JM, Sagovsky R. Detection of Postnatal Depression: Development of the 10-item Edinburgh Postnatal Depression Scale. British Journal of Psychiatry. 1987;150(6):782-6.

23. Bowen E, Heron J, Waylen A, Wolke D. Domestic violence risk during and after pregnancy: findings from a British longitudinal study. BJOG : an international journal of obstetrics and gynaecology. 2005;112(8):1083-9.

24. Rubin DB. Multiple imputation for nonresponse in surveys: John Wiley & Sons; 2004.

**Supplementary Table** **1: Comparison of characteristics for participants who did versus did not attend age 17 and/or age 24 clinics**

|  | Attended at age 17 and/or age 24, *n*=6087 | Missing data, *n* (%) |  | Did not attend at age 17 and/or age 24, *n*=9559 | Missing  data, *n* (%) | Test statistic comparing characteristics | *p* |
| --- | --- | --- | --- | --- | --- | --- | --- |
| Sex, n (%) | 3487 female (57.3%)  2595 male (42.7%) | 5  (0.1%) |  | 3861 female (43.1%)  5100 male (56.9%) | 598 (6.3%) | χ^2^ = 294.3 | <0.001 |
| Ethnicity, n (%) | 5095 white (95.6%)  232 non-white (4.4%) | 760 (12.5%) |  | 6432 white (94.4%)  381 non-white (5.6%) | 2746 (28.7%) | χ^2^ = 9.5 | 0.002 |
| Home ownership status at 8 weeks gestation, n (%) | 4557 own home or mortgage (86.0%)  743 rent or other (14.0%) | 787 (12.9%) |  | 5329 own home or mortgage (69.0%)  2400 rent or other (31.0%) | 1830 (19.1%) | χ^2^ = 498.3 | <0.001 |
| Highest parental social class at 32 weeks gestation, n (%) | 4478 non-manual (86.7%)  689 manual (13.3%) | 920 (15.1%) |  | 4836 non-manual (75.6%)  1559 manual (24.4%) | 3164 (33.1%) | χ^2^ = 222.5 | <0.001 |
| Highest maternal educational qualification at 32 weeks gestation, n (%) | 4339 O-level or higher (80.3%)  1068 less than O-level (19.7%) | 680 (11.2%) |  | 4398 O-level or higher (62.1%)  2688 less than O-level (37.9%) | 2473 (25.9%) | χ^2^ = 482.2 | <0.001 |
| Take-home family income at age 8 years, n (%) | 2326 £400+ per week (55.7%)  1852 <£400 per week (44.3%) | 1909 (31.4%) |  | 1289 £400+ per week (44.1%)  1632 <£400 per week (55.9%) | 6638 (69.4%) | χ^2^ = 91.7 | <0.001 |
| IQ score at age 8 years, mean (SD) | 106.6 (16.3) | 1369 (22.5%) |  | 99.3 (15.9) | 7329 (76.7%) | t = -18.7 | <0.001 |

IQR: interquartile range; SD: standard deviation.

Data for highest parental social class, take-home family income, home ownership status and highest maternal qualification were collected by questionnaires completed by the mother prior to birth of the participant.

O-levels (Ordinary levels) are the standard examinations taken by students in the United Kingdom at approximately age 16.

**Supplementary Table** **2: Comparison of characteristics for participants with and without polyunsaturated fatty acid data available at age 17 years (total age 17 sample *n*=5215)**

|  | PUFA data available, *n*=3166 | Missing data, *n* (%) |  | PUFA data not available, *n*=2049 | Missing data, *n* (%) | Test statistic comparing characteristics | *p* |
| --- | --- | --- | --- | --- | --- | --- | --- |
| Age in years at attendance of age 17 clinic, mean (SD) | 17.8 (0.4) | 0 (0%) |  | 17.9 (0.5) | 0 (0%) | t = 7.8 | <0.001 |
| Sex, n (%) | ≥52.0% female  ≤48.0% male | ≤0.2%* |  | ≥63.1% female  ≤36.9% male | ≤0.2%* | χ^2^ = 61.8 | <0.001 |
| Ethnicity, n (%) | 2718 white (95.6%)  125 non-white (4.4%) | 323 (10.2%) |  | 1707 white (95.6%)  79 non-white (4.4%) | 263 (12.8%) | χ^2^ < 0.01 | 0.966 |
| BMI in kg/m^2^ at age 17 years, mean (SD) | 22.7 (3.9) | 82 (2.6%) |  | 23.1 (4.6) | 71 (3.5%) | t = 2.9 | 0.004 |
| Daily smoker at age 17 years, n (%) | 2316 no (87.6%)  328 yes (12.4%) | 522 (16.5%) |  | 1359 no (87.4%)  196 yes (12.6%) | 494 (24.1%) | χ^2^ = 0.03 | 0.851 |
| Average number of cigarettes smoked per day if daily smoker, median (IQR) | 9.5 (10) | 522 (16.5%) |  | 10 (6) | 494 (24.1%) | z = 0.337 | 0.736 |
| AUDIT score at age 17 years, median (IQR) | 7 (6) | 718 (22.7%) |  | 7 (6) | 646 (31.5%) | z = 0.3 | 0.743 |
| Regular cannabis use^a^ at age 17 years, n (%) | 2416 no (92.4%)  200 yes (7.6%) | 550 (17.4%) |  | 1435 no (93.5%)  100 yes (6.5%) | 514 (25.1%) | χ^2^ = 1.8 | 0.174 |
| Psychotic disorder at age 17 years, n (%) | 2943 no (98.5%)  44 yes (1.5%) | 179 (5.7%) |  | 1696 no (98.0%)  35 yes (2.0%) | 318 (15.5%) | χ^2^ = 2.0 | 0.157 |
| Moderate/ severe depressive disorder at age 17 years, n (%) | 2774 no (95.8%)  123 yes (4.3%) | 269 (8.5%) |  | 1562 no (93.8%)  104 yes (6.2%) | 383 (18.7%) | χ^2^ = 8.9 | 0.003 |
| Generalised anxiety disorder at age 17 years, n (%) | 2741 no (94.6%)  156 yes (5.4%) | 269 (8.5%) |  | 1559 no (93.6%)  107 yes (6.4%) | 383 (18.7%) | χ^2^ = 2.1 | 0.148 |
| Home ownership status at 8 weeks gestation, n (%) | 2433 own home or mortgage (86.8%)  369 rent or other (13.2%) | 364 (11.5%) |  | 1548 own home or mortgage (85.9%)  255 rent or other (14.1%) | 246 (12.0%) | χ^2^ = 0.9 | 0.346 |
| Highest parental social class at 32 weeks gestation, n (%) | 2416 non-manual (87.4%)  349 manual (12.6%) | 401 (12.7%) |  | 1483 non-manual (85.8%)  245 manual (14.2%) | 321 (15.7%) | χ^2^ = 2.2 | 0.134 |
| Highest maternal educational qualification at 32 weeks gestation, n (%) | 2366 O-level or higher (82.2%)  514 less than O-level (17.8%) | 286 (9.0%) |  | 1428 O-level or higher (78.3%)  395 less than O-level (21.7%) | 226 (11.0%) | χ^2^ = 10.4 | 0.001 |
| Take-home family income at age 8 years, n (%) | 1337 £400+ per week (57.3%)  995 <£400 per week (42.7%) | 834 (26.3%) |  | 752 £400+ per week (46.2%)  752 <£400 per week (53.8%) | 652 (31.8%) | χ^2^ = 4.4 | 0.037 |
| IQ score at age 8 years, mean (SD) | 108.0 (16.5) | 546 (17.2%) |  | 105.2 (16.1) | 430 (21.0%) | t = -5.4 | <0.001 |

^a^ Regular cannabis use at age 17 was defined as using cannabis more than once monthly.
* Data suppressed due to small cell counts
AUDIT: Alcohol Use Disorders Identification Test; IQR: interquartile range; PUFA: polyunsaturated fatty acid; SD: standard deviation.

**Supplementary Table** **3: Comparison of characteristics for participants with and without polyunsaturated fatty acid data available at age 24 years (total age 24 sample *n*=4019)**

|  | PUFA data available, *n*=3257 | Missing data, *n* (%) |  | PUFA data not available, *n*=762 | Missing data, *n* (%) | Test statistic comparing characteristics | *p* |
| --- | --- | --- | --- | --- | --- | --- | --- |
| Age in years at attendance of age 17 clinic, mean (SD) | 24.0 (0.9) | 0 (0%) |  | 24.0 (0.8) | 0 (0%) | t = 0.5 | 0.596 |
| Sex, n (%) | ≥60.2% female  ≤39.8% male | ≤0.2%* |  | 553 female (72.6%)  209 male (27.4%) | 0 (0%) | χ^2^ = 40.4 | <0.001 |
| Ethnicity, n (%) | 2781 white (96.3%)  107 non-white (3.7%) | 369 (11.3%) |  | 642 white (94.7%)  36 non-white (5.3%) | 84 (11.0%) | χ^2^ = 3.7 | 0.055 |
| BMI in kg/m^2^ at age 24 years, mean (SD) | 24.7 (4.9) | 33 (1.0%) |  | 25.8 (5.8) | 11 (1.4%) | t = 4.5 | <0.001 |
| Daily smoker at age 24 years, n (%) | 2827 no (88.2%)  379 yes (11.8%) | 51 (1.6%) |  | 641 no (85.2%)  111 yes (14.8%) | 10 (1.3%) | χ^2^ = 4.9 | 0.028 |
| Average number of cigarettes smoked per day if daily smoker, median (IQR) | 10 (7) | 51 (1.6%) |  | 10 (7) | 10 (1.3%) | z = <0.1 | 0.987 |
| AUDIT-C score at age 24 years, median (IQR) | 5 (3) | 71 (2.2%) |  | 5 (4) | 19 (2.5%) | z = -4.0 | <0.001 |
| Regular cannabis use^a^ at age 24 years, n (%) | 3016 no (93.5%)  209 yes (6.5%) | 32 (0.1%) |  | ≥94.8% no  ≤5.2% yes | ≤0.7%* | χ^2^ = 1.8 | 0.184 |
| Psychotic disorder at age 24 years, n (%) | 3127 no (98.8%)  37 yes (1.4%) | 93 (2.9%) |  | 715 no (98.6%)  10 yes (1.4%) | 37 (4.9%) | χ^2^ = 0.2 | 0.641 |
| Moderate/ severe depressive disorder at age 24 years, n (%) | 2986 no (92.8%)  230 yes (7.2%) | 41 (1.3%) |  | 676 no (90.1%)  74 yes (9.9%) | 12 (1.6%) | χ^2^ = 6.3 | 0.012 |
| Generalised anxiety disorder at age 24 years, n (%) | 2907 no (90.6%)  301 yes (9.4%) | 49 (1.5%) |  | 664 no (88.7%)  85 yes (11.4%) | 13 (1.7%) | χ^2^ = 2.7 | 0.103 |
| Home ownership status at 8 weeks gestation, n (%) | 2525 own home or mortgage (88.4%)  331 rent or other (11.6%) | 401 (12.3%) |  | 554 own home or mortgage (84.5%)  102 rent or other (15.5%) | 106 (13.9%) | χ^2^ = 7.7 | 0.005 |
| Highest parental social class at 32 weeks gestation, n (%) | 2502 non-manual (88.9%)  314 manual (11.1%) | 441 (13.5%) |  | 563 non-manual (86.4%)  89 manual (13.6%) | 110 (14.4%) | χ^2^ = 3.2 | 0.073 |
| Highest maternal educational qualification at 32 weeks gestation, n (%) | 2442 O-level or higher (83.6%)  480 less than O-level (16.4%) | 335 (10.3%) |  | 554 O-level or higher (80.4%)  135 less than O-level (19.6%) | 73 (9.6%) | χ^2^ = 4.0 | 0.047 |
| Take-home family income at age 8 years, n (%) | 1372 £400+ per week (58.9%)  958 <£400 per week (41.1%) | 927 (28.5%) |  | 301 £400+ per week (56.3%)  234 <£400 per week (43.7%) | 227 (29.8%) | χ^2^ = 1.2 | 0.267 |
| IQ score at age 8, mean (SD) | 108.7 (15.8) | 653 (20.0%) |  | 104.4 (16.0) | 146 (19.2%) | t = -6.0 | <0.001 |

^a^ Regular cannabis use at age 24 was defined as using cannabis weekly or daily.
* Data suppressed due to small cell counts.
AUDIT-C: Alcohol Use Disorders Identification Test – Concise; IQR: interquartile range; PUFA: polyunsaturated fatty acid; SD: standard deviation.

**Supplementary Table** **4: Characteristics of age 17 sample according to specific mental disorder at age 17 years**

|  | | **Psychotic disorder** | | | | **Moderate/severe depressive disorder** | | | | **Generalised anxiety disorder** | | | |
| --- | --- | --- | --- | --- | --- | --- | --- | --- | --- | --- | --- | --- | --- |
|  |  | **Yes  *n*=79** | **No  *n*=4639** | ***p*** | **Missing data, n (%)** | **Yes  *n*=227** | **No  *n*=4336** | ***p*** | **Missing data, n (%)** | **Yes  *n*=263** | **No  *n*=4300** | ***p*** | **Missing data, n (%)** |
| **Age in years, mean (SD)** | | 17.8 (0.5) | 17.8 (0.4) | 0.262 | 0 (0%) | 17.8 (0.4) | 17.8 (0.4) | 0.850 | 0 (0%) | 17.8 (0.4) | 17.8 (0.4) | 0.293 | 0 (0%) |
| **Sex*, n (%)** | Female | ≥78.5% | ≥56.2% | <0.001 | ≤0.2% | ≥77.1% | ≥55.2% | <0.001 | ≤0.2% | ≥74.8% | ≥55.2% | <0.001 | ≤0.2% |
|  | Male | ≤21.5% | ≤43.8% |  |  | ≤22.9%) | ≤44.8% |  |  | ≤25.2% | ≤44.8% |  |  |
| **Ethnicity, n (%)** | White | 63 (92.6%) | 3982 (95.8%) | 0.203 | 493 (10.4%) | 186 (93.5%) | 3729 (95.8%) | 0.112 | 472 (10.3%) | 227 (95.8%) | 3688 (95.7%) | 0.948 | 472 (10.3%) |
|  | Non-white | 5  (7.4%) | 175 (4.2%) |  |  | 13 (6.5%) | 163 (4.2%) |  |  | 10 (4.2%) | 166 (4.3%) |  |  |
| **BMI in kg/m^2^ at age 17 years, mean (SD)** | | 23.2 (4.5) | 22.8 (4.2) | 0.474 | 73 (1.5%) | 23.4 (5.3) | 22.8 (4.1) | 0.079 | 85 (1.9%) | 22.7 (4.4) | 22.8 (4.2) | 0.631 | 85 (1.9%) |
| **Daily smoker at age 17 years, n (%)** | No | 42 (66.7%) | 3499 (88.0%) | <0.001 | 680 (14.4%) | 126 (69.2%) | 3390 (88.5%) | <0.001 | 552 (12.1%) | 178 (80.5%) | 3338 (88.1%) | 0.001 | 552 (12.1%) |
|  | Yes | 21 (33.3%) | 476 (12.0%) |  |  | 56 (30.8%) | 439 (11.5%) |  |  | 43 (19.5%) | 452 (11.9%) |  |  |
| **Average number of cigarettes smoked per day if daily smoker, median (IQR)** | | 10 (10) | 10 (8) | 0.544 | 680 (14.4%) | 10 (8.5) | 8 (8) | 0.008 | 552 (12.1%) | 8 (10) | 10 (9.5) | 0.811 | 552 (12.1%) |
| **AUDIT score at age 17 years, median (IQR)** | | 8.5 (7.5) | 7 (6) | 0.081 | 1012 (21.4%) | 9 (7) | 7 (6) | <0.001 | 884 (19.4%) | 8 (6) | 7 (6) | 0.003 | 884 (19.4%) |
| **Regular cannabis use^a^ at age 17 years, n (%)** | No | 51 (82.3%) | 3655 (93.0%) | 0.001 | 724 (15.3%) | 149 (83.2%) | 3533 (93.3%) | <0.001 | 598 (13.1%) | 185 (85.2%) | 3497 (93.3%) | <0.001 | 598 (13.1%) |
|  | Yes | 11 (17.7%) | 277 (7.0%) |  |  | 30 (16.8%) | 253 (6.7%) |  |  | 32 (14.8%) | 251 (6.7%) |  |  |
| **Home ownership status at 8 weeks gestation, n (%)** | Own home or mortgage | 47 (68.1%) | 3600 (87.4%) | <0.001 | 531 (11.3%) | 160 (81.6%) | 3378 (87.6%) | 0.015 | 509 (11.2%) | 194 (82.5%) | 3344 (87.6%) | 0.025 | 509 (11.2%) |
|  | Rent or other | 22 (31.9%) | 518 (12.6%) |  |  | 36 (18.4%) | 480 (12.4%) |  |  | 41 (17.5%) | 475 (12.4%) |  |  |
| **Highest parental social class at 32 weeks gestation, n (%)** | Non-manual | 47 (77.0%) | 3543 (87.4%) | 0.016 | 603 (12.8%) | 164 (86.3%) | 3311 (87.3%) | 0.680 | 582 (12.8%) | 201 (87.8%) | 3274 (87.3%) | 0.821 | 582 (12.8%) |
|  | Manual | 14 (23.0%) | 511 (12.6%) |  |  | 26 (13.7%) | 480 (12.7%) |  |  | 28 (12.2%) | 478 (12.7%) |  |  |
| **Highest maternal educational qualification at 32 weeks gestation, n (%)** | O-level or higher | 51 (71.8%) | 3432 (81.3%) | 0.042 | 427 (9.1%) | 170 (82.9%) | 3204 (81.3%) | 0.550 | 415 (9.1%) | 201 (83.4%) | 3173 (81.2%) | 0.397 | 415 (9.1%) |
|  | Less than O-level | 20 (28.2%) | 788 (18.7%) |  |  | 35 (17.1%) | 739 (18.7%) |  |  | 40 (16.6%) | 734 (18.8%) |  |  |
| **Take-home family income at age 8 years, n (%)** | £400+ per week | 22 (47.8%) | 1926 (56.8%) | 0.222 | 1282 (27.2%) | 77 (47.5%) | 1806 (56.9%) | 0.019 | 1229 (26.9%) | 100 (56.5%) | 1783 (56.5%) | 0.996 | 1229 (26.9%) |
|  | <£400 per week | 24 (52.3%) | 1464 (43.2%) |  |  | 85 (52.5%) | 1366 (43.1%) |  |  | 77 (43.5%) | 1374 (43.5%) |  |  |
| **IQ score at age 8, mean (SD)** | | 104.3 (15.8) | 107.4 (16.3) | 0.150 | 804 (17.0%) | 107.1 (15.6) | 107.4 (16.0) | 0.766 | 764 (16.7%) | 105.9 (15.8) | 10.7.5 (16.0) | 0.167 | 764 (16.7%) |

AUDIT: Alcohol Use Disorders Identification Test; IQR: interquartile range; DHA: decosahexaenoic acid; n-6: omega-6; n-3: omega-3; PUFA: polyunsaturated fatty acid; SD: standard deviation; N/A: not applicable. Exact numbers are not given for cell counts with less than 5 participants to avoid statistical disclosure.

^a^ Regular cannabis use at age 17 was defined as using cannabis more than once monthly.
* Exact numbers not shown due to small cell counts for missing data.

**Supplementary Table** **5: Characteristics of age 24 sample according to specific mental disorder at age 24 years**

|  | | **Psychotic disorder** | | | | **Moderate/severe depressive disorder** | | | | **Generalised anxiety disorder** | | | |
| --- | --- | --- | --- | --- | --- | --- | --- | --- | --- | --- | --- | --- | --- |
|  |  | **Yes  *n*=47** | **No  *n*=3842** | ***p*** | **Missing data, n (%)** | **Yes  *n*=304** | **No  *n*=3662** | ***p*** | **Missing data, n (%)** | **Yes  *n*=386** | **No  *n*=3571** | ***p*** | **Missing data, n (%)** |
| **Age in years, mean (SD)** | | 24.1 (0.9) | 24.0 (0.8) | 0.571 | 0 (0%) | 24.1 (0.8) | 24.0 (0.8) | 0.182 | 0 (0%) | 24.1 (0.8) | 24.0 (0.8) | 0.909 | 0 (0%) |
| **Sex*, n (%)** | Female | ≥74.5% | ≥62.3% | 0.088 | ≤0.2% | ≥77.3% | ≥61.4% | <0.001 | ≤0.2% | ≥75.1% | ≥61.3% | <0.001 | ≤0.2% |
|  | Male | ≤25.5% | ≤37.7% |  |  | ≤22.7% | ≤38.6% |  |  | ≤24.9% | ≤38.7% |  |  |
| **Ethnicity*, n (%)** | White | ≥87.5% | ≥96.1% | 0.251 | 441 (11.3%) | 253 (95.1%) | 3127 (96.1%) | 0.430 | 446 (11.2%) | 324 (96.1%) | 3047 (96.0%) | 0.898 | 446 (11.3%) |
|  | Non-white | ≤12.5% | ≤3.9% |  |  | 13 (4.9%) | 127 (3.9%) |  |  | 13 (3.9%) | 127 (4.0%) |  |  |
| **BMI in kg/m^2^ at age 24 years, mean (SD)** | | 26.7 (7.5) | 24.9 (5.0) | 0.098 | 37 (1.0%) | 26.4 (6.9) | 24.8 (4.9) | <0.001 | 40 (1.0%) | 25.6 (6.5) | 24.8 (4.9) | 0.019 | 40 (1.0%) |
| **Daily smoker at age 24 years, n (%)** | No | 30 (65.2%) | 3347 (88.3%) | <0.001 | 51 (1.3%) | 237 (78.5%) | 3219 (88.3%) | <0.001 | 51 (1.3%) | 302 (78.7%) | 3146 (88.6%) | <0.001 | 21 (0.5%) |
|  | Yes | 16 (34.8%) | 445 (11.7%) |  |  | 65 (21.5%) | 425 (11.7%) |  |  | 82 (21.3%) | 406 (11.4%) |  |  |
| **Average number of cigarettes smoked per day if daily smoker, median (IQR)** | | 10 (8.5) | 9 (7) | 0.105 | 51 (1.3%) | 9 (7) | 10 (7) | 0.920 | 51 (1.3%) | 10 (7) | 9.5 (7) | 0.910 | 21 (0.5%) |
| **AUDIT-C score at age 24 years, median (IQR)** | | 4 (4) | 5 (3) | 0.004 | 77 (2.0%) | 5 (4) | 5 (3) | 0.057 | 50 (1.3%) | 5 (4) | 5 (3) | 0.060 | 49 (1.2%) |
| **Regular cannabis use^a^* at age 24 years, n (%)** | No | 41 (89.1%) | 3577 (93.8%) | 0.189 | 31 (0.8%) | ≥88.5% | ≥94.2% | <0.001 | ≤0.2% | ≥88.3% | ≥94.3% | <0.001 | ≤0.2% |
|  | Yes | 5 (10.9%) | 235 (6.2%) |  |  | ≤11.5% | ≤5.8% |  |  | ≤11.7% | ≤5.7% |  |  |
| **Home ownership status at 8 weeks gestation, n (%)** | Own home or mortgage | 30 (75.0%) | 2957 (88.2%) | 0.011 | 495 (12.7%) | 209 (79.5%) | 2836 (88.5%) | <0.001 | 420 (10.6%) | 274 (82.5%) | 2762 (88.3%) | 0.002 | 496 (12.5%) |
|  | Rent or other | 10 (25.0%) | 397 (11.8%) |  |  | 54 (20.5%) | 370 (11.5%) |  |  | 58 (17.5%) | 367 (11.7%) |  |  |
| **Highest parental social class at 32 weeks gestation*, n (%)** | Non-manual | ≥89.2% | ≥88.5% | 0.900 | 532 (13.7%) | 218 (84.8%) | 2811 (88.7%) | 0.060 | 541 (13.6%) | 278 (85.3%) | 2740 (88.7%) | 0.066 | 542 (13.7%) |
|  | Manual | ≤10.8% | ≤11.5% |  |  | 39 (15.2%) | 357 (11.3%) |  |  | 48 (14.7%) | 349 (11.3%) |  |  |
| **Highest maternal educational qualification at 32 weeks gestation, n (%)** | O-level or higher | 33 (80.5%) | 2878 (83.5%) | 0.610 | 400 (10.3%) | 226 (83.1%) | 2734 (83.0%) | 0.970 | 323 (8.1%) | 280 (81.4%) | 2670 (83.1%) | 0.417 | 401 (10.1%) |
|  | Less than O-level | 8 (19.5%) | 570 (16.5%) |  |  | 46 (16.9%) | 560 (17.0%) |  |  | 64 (18.6%) | 542 (16.9%) |  |  |
| **Take-home family income at age 8 years, n (%)** | £400+ per week | 13 (48.2%) | 1607 (58.6%) | 0.273 | 1119 (28.8%) | 105 (52.2%) | 1549 (58.9%) | 0.064 | 1059 (26.7%) | 139 (53.7%) | 1512 (58.9%) | 0.102 | 1132 (28.6%) |
|  | <£400 per week | 14 (51.8%) | 1136 (41.4%) |  |  | 96 (47.8%) | 1080 (41.1%) |  |  | 120 (46.3%) | 1054 (41.1%) |  |  |
| **IQ score at age 8, mean (SD)** | | 99.9 (13.8) | 108.2 (15.8) | 0.003 | 765 (19.7%) | 107.4 (16.8) | 108.1 (15.8) | 0.518 | 787 (19.8%) | 107.8 (15.8) | 108.1 (15.9) | 0.798 | 787 (19.9%) |

AUDIT-C: Alcohol Use Disorders Identification Test – Concise; IQR: interquartile range; DHA: decosahexaenoic acid; n-6: omega-6; n-3: omega-3; PUFA: polyunsaturated fatty acid; SD: standard deviation; N/A: not applicable. Exact numbers are not given for cell counts with less than 5 participants to avoid statistical disclosure.

^a^ Regular cannabis use at age 24 was defined as using cannabis weekly or daily.

* Exact numbers not shown due to small cell counts.

**Supplementary Table 6: Effect estimates for associations between change in plasma polyunsaturated fatty acid measures from 17 to 24 years and odds of incident mental disorders at 24 years**

| **Outcome (*n* with vs. without outcome)** | **Exposure** | **Crude** | | | **Adjusted** | | |
| --- | --- | --- | --- | --- | --- | --- | --- |
|  |  | **Odds ratio** | **95% CI** | ***p*** | **Odds ratio** | **95% CI** | ***p*** |
| **Psychotic disorder** (20 vs. 2754) | Change in total n-6 PUFAs | 1.28 | 0.75 – 2.16 | 0.362 | 1.23 | 0.72 – 2.09 | 0.444 |
|  | Change in total n-3 PUFAs | 1.18 | 0.70 – 1.97 | 0.531 | 1.18 | 0.71 – 1.98 | 0.520 |
|  | Change in n-6:n-3 ratio | 1.16 | 0.65 – 2.04 | 0.618 | 1.09 | 0.64 – 1.86 | 0.750 |
|  | Change in DHA % total fatty acids | 0.97 | 0.59 – 1.57 | 0.888 | 1.03 | 0.63 – 1.69 | 0.900 |
| **Moderate/ severe depressive disorder** (157 vs. 2505) | Change in total n-6 PUFAs | 0.97 | 0.76 – 1.23 | 0.792 | 0.98 | 0.77 – 1.24 | 0.876 |
|  | Change in total n-3 PUFAs | 0.85 | 0.68 – 1.06 | 0.152 | 0.87 | 0.69 – 1.08 | 0.208 |
|  | Change in n-6:n-3 ratio | 1.21 | 0.92 – 1.60 | 0.166 | 1.18 | 0.90 – 1.53 | 0.227 |
|  | Change in DHA % total fatty acids | 0.94 | 0.76 – 1.16 | 0.553 | 0.96 | 0.78 – 1.19 | 0.720 |
| **Generalised anxiety disorder** (205 vs. 2423) | Change in total n-6 PUFAs | 0.79 | 0.66 – 0.95 | 0.011 | 0.83 | 0.69 – 1.00 | 0.045 |
|  | Change in total n-3 PUFAs | 0.85 | 0.71 – 1.00 | 0.057 | 0.88 | 0.74 – 1.04 | 0.133 |
|  | Change in n-6:n-3 ratio | 1.02 | 0.84 – 1.25 | 0.81- | 1.02 | 0.84 – 1.24 | 0.837 |
|  | Change in DHA % total fatty acids | 0.98 | 0.83 – 1.16 | 0.794 | 0.97 | 0.83 – 1.15 | 0.759 |

BMI: body mass index; CI: confidence interval; DHA: docosahexaenoic acid; n-6: omega-6; n-3: omega-3; PUFA: polyunsaturated fatty acid.

Change in PUFA measures were converted to z-scores such that the odds ratios may be interpreted as per standard deviation increase in change in PUFA measure between 17 and 24 years. Covariates in adjusted models included sex; change in BMI between 17 and 24 years; and change in average number of cigarettes smoked per day between 17 and 24 years.

**Supplementary Table** **7: Odds ratios for cross-sectional associations between PUFA measures and mental disorders at age 17 years in models with additional adjustment for further potential confounding factors**

| **Outcome** | **Exposure** | **Base model: adjusted for age, sex, BMI, cigarettes at age 17 years** | **Base + AUDIT score at age 17 years** | **Base + regular cannabis use^a^ at age 17 years** | **Base +  home ownership status at 8 weeks gestation** | **Base + parental social class at 32 weeks gestation** | **Base + maternal education at 32 weeks gestation** | **Base +**  **family income at age 8 years** | **Base +**  **IQ at age 8 years** |
| --- | --- | --- | --- | --- | --- | --- | --- | --- | --- |
| **Psychotic disorder** | Total n-6 PUFAs | 0.83 | 0.83 | 0.82 | 0.84 | 0.83 | 0.83 | 0.83 | 0.83 |
|  | Total n-3 PUFAs | 0.89 | 0.89 | 0.89 | 0.90 | 0.90 | 0.90 | 0.90 | 0.90 |
|  | n-6:n-3 ratio | 0.96 | 0.96 | 0.96 | 0.95 | 0.95 | 0.95 | 0.95 | 0.95 |
|  | DHA % total fatty acids | 0.77 | 0.77 | 0.77 | 0.80 | 0.79 | 0.78 | 0.79 | 0.78 |
| **Moderate/ severe depressive disorder** | Total n-6 PUFAs | 0.97 | 0.97 | 0.96 | 0.97 | 0.97 | 0.97 | 0.97 | 0.96 |
|  | Total n-3 PUFAs | 0.98 | 0.98 | 0.97 | 0.98 | 0.97 | 0.97 | 0.99 | 0.97 |
|  | n-6:n-3 ratio | 0.99 | 0.99 | 0.98 | 0.99 | 0.99 | 1.00 | 0.98 | 0.99 |
|  | DHA % total fatty acids | 0.95 | 0.95 | 0.95 | 0.95 | 0.95 | 0.93 | 0.97 | 0.93 |
| **Generalised anxiety disorder** | Total n-6 PUFAs | 0.91 | 0.92 | 0.90 | 0.91 | 0.91 | 0.91 | 0.91 | 0.91 |
|  | Total n-3 PUFAs | 1.03 | 1.03 | 1.02 | 1.03 | 1.03 | 1.02 | 1.03 | 1.04 |
|  | n-6:n-3 ratio | 0.89 | 0.89 | 0.89 | 0.89 | 0.89 | 0.90 | 0.89 | 0.88 |
|  | DHA % total fatty acids | 1.02 | 1.02 | 1.02 | 1.03 | 1.02 | 1.01 | 1.02 | 1.03 |

AUDIT: Alcohol Use Disorders Identification Test; CI: confidence interval; DHA: docosahexaenoic acid; n-6: omega-6; n-3: omega-3; PUFA: polyunsaturated fatty acid.
^a^ Regular cannabis use at age 17 was defined as using cannabis more than once monthly.

PUFA measures were converted to z-scores such that the odds ratios may be interpreted as per standard deviation increase in the PUFA measure.

**Supplementary Table** **8: Odds ratios for cross-sectional associations between PUFA measures and mental disorders at age 24 years in models with additional adjustment for further potential confounding factors**

| **Outcome** | **Exposure** | **Base model: adjusted for age, sex, BMI, cigarettes at age 24 years** | **Base + AUDIT-C score at age 24 years** | **Base + regular cannabis use^a^ at age 24 years** | **Base +  home ownership status at 8 weeks gestation** | **Base + parental social class at 32 weeks gestation** | **Base + maternal education at 32 weeks gestation** | **Base +**  **family income at age 8 years** | **Base +**  **IQ at age 8 years** |
| --- | --- | --- | --- | --- | --- | --- | --- | --- | --- |
| **Psychotic disorder** | Total n-6 PUFAs | 1.07 | 1.08 | 1.07 | 1.07 | 1.07 | 1.07 | 1.07 | 1.07 |
|  | Total n-3 PUFAs | 0.71 | 0.75 | 0.71 | 0.73 | 0.71 | 0.71 | 0.72 | 0.73 |
|  | n-6:n-3 ratio | 1.43 | 1.41 | 1.43 | 1.41 | 1.43 | 1.43 | 1.43 | 1.40 |
|  | DHA % total fatty acids | 0.60 | 0.63 | 0.60 | 0.61 | 0.60 | 0.60 | 0.61 | 0.62 |
| **Moderate/ severe depressive disorder** | Total n-6 PUFAs | 1.00 | 1.00 | 1.00 | 1.00 | 1.00 | 1.00 | 1.00 | 1.00 |
|  | Total n-3 PUFAs | 0.87 | 0.87 | 0.87 | 0.88 | 0.87 | 0.87 | 0.87 | 0.87 |
|  | n-6:n-3 ratio | 1.18 | 1.18 | 1.18 | 1.17 | 1.18 | 1.18 | 1.18 | 1.18 |
|  | DHA % total fatty acids | 0.87 | 0.87 | 0.87 | 0.88 | 0.87 | 0.87 | 0.87 | 0.87 |
| **Generalised anxiety disorder** | Total n-6 PUFAs | 0.98 | 0.98 | 0.98 | 0.98 | 0.98 | 0.98 | 0.98 | 0.98 |
|  | Total n-3 PUFAs | 0.90 | 0.90 | 0.90 | 0.90 | 0.90 | 0.90 | 0.90 | 0.90 |
|  | n-6:n-3 ratio | 1.14 | 1.13 | 1.14 | 1.13 | 1.14 | 1.14 | 1.13 | 1.14 |
|  | DHA % total fatty acids | 0.89 | 0.90 | 0.90 | 0.90 | 0.89 | 0.89 | 0.90 | 0.89 |

AUDIT-C: Alcohol Use Disorders Identification Test – Concise ; CI: confidence interval; DHA: docosahexaenoic acid; n-6: omega-6; n-3: omega-3; PUFA: polyunsaturated fatty acid. ^a^ Regular cannabis use at age 24 was defined as using cannabis weekly or daily.

PUFA measures were converted to z-scores such that the odds ratios may be interpreted as per standard deviation increase in the PUFA measure.

**Supplementary Table** **9: Odds ratios for longitudinal associations between PUFA measures at age 17 years and incident mental disorders at age 24 years in models with additional adjustment for further potential confounding factors**

| **Outcome** | **Exposure** | **Base model: adjusted for age, sex, BMI, cigarettes at age 17 years** | **Base + AUDIT score at age 17 years** | **Base + regular cannabis use^a^ at age 17 years** | **Base +  home ownership status at 8 weeks gestation** | **Base + parental social class at 32 weeks gestation** | **Base + maternal education at 32 weeks gestation** | **Base +**  **family income at age 8 years** | **Base +**  **IQ at age 8 years** |
| --- | --- | --- | --- | --- | --- | --- | --- | --- | --- |
| **Psychotic disorder** | Total n-6 PUFAs | 1.06 | 1.06 | 1.06 | 1.07 | 1.06 | 1.05 | 1.08 | 1.06 |
|  | Total n-3 PUFAs | 0.65 | 0.66 | 0.66 | 0.66 | 0.65 | 0.67 | 0.68 | 0.68 |
|  | n-6:n-3 ratio | 1.42 | 1.42 | 1.40 | 1.42 | 1.42 | 1.39 | 1.38 | 1.36 |
|  | DHA % total fatty acids | 0.44 | 0.44 | 0.45 | 0.46 | 0.44 | 0.45 | 0.47 | 0.48 |
| **Moderate/ severe depressive disorder** | Total n-6 PUFAs | 0.98 | 0.98 | 0.98 | 0.99 | 0.98 | 0.98 | 0.99 | 0.98 |
|  | Total n-3 PUFAs | 0.98 | 0.98 | 0.98 | 0.99 | 0.99 | 0.97 | 0.99 | 0.98 |
|  | n-6:n-3 ratio | 1.02 | 1.03 | 1.02 | 1.02 | 1.02 | 1.04 | 1.02 | 1.02 |
|  | DHA % total fatty acids | 0.89 | 0.89 | 0.89 | 0.90 | 0.89 | 0.88 | 0.90 | 0.89 |
| **Generalised anxiety disorder** | Total n-6 PUFAs | 1.14 | 1.15 | 1.14 | 1.14 | 1.14 | 1.14 | 1.14 | 1.14 |
|  | Total n-3 PUFAs | 0.98 | 0.98 | 0.98 | 0.98 | 0.98 | 0.97 | 0.98 | 0.97 |
|  | n-6:n-3 ratio | 1.14 | 1.14 | 1.13 | 1.14 | 1.14 | 1.15 | 1.14 | 1.15 |
|  | DHA % total fatty acids | 0.89 | 0.88 | 0.88 | 0.89 | 0.89 | 0.88 | 0.89 | 0.88 |

AUDIT: Alcohol Use Disorders Identification Test; CI: confidence interval; DHA: docosahexaenoic acid; n-6: omega-6; n-3: omega-3; PUFA: polyunsaturated fatty acid.
^a^ Regular cannabis use at age 17 was defined as using cannabis more than once monthly.

PUFA measures were converted to z-scores such that the odds ratios may be interpreted as per standard deviation increase in the PUFA measure.

**Supplementary Table** **10: Odds ratios for longitudinal associations between change in PUFA measures from age 17 to age 24 years and incident mental disorders at age 24 years in models with additional adjustment for further potential confounding factors**

| **Outcome** | **Exposure** | **Base model: adjusted for sex, change in BMI, change in cigarettes** | **Base + AUDIT score at age 17 years** | **Base + regular cannabis use^a^ at age 17 years** | **Base +  home ownership status at 8 weeks gestation** | **Base + parental social class at 32 weeks gestation** | **Base + maternal education at 32 weeks gestation** | **Base +**  **family income at age 8 years** | **Base +**  **IQ at age 8 years** |
| --- | --- | --- | --- | --- | --- | --- | --- | --- | --- |
| **Psychotic disorder** | Change in total n-6 PUFAs | 1.23 | 1.21 | 1.24 | 1.23 | 1.23 | 1.23 | 1.25 | 1.22 |
|  | Change in total n-3 PUFAs | 1.18 | 1.16 | 1.18 | 1.21 | 1.18 | 1.16 | 1.18 | 1.16 |
|  | Change in n-6:n-3 ratio | 1.09 | 1.10 | 1.11 | 1.07 | 1.09 | 1.11 | 1.10 | 1.11 |
|  | Change in DHA % total fatty acids | 1.03 | 1.02 | 1.03 | 1.04 | 1.03 | 1.02 | 1.01 | 0.99 |
| **Moderate/ severe depressive disorder** | Change in total n-6 PUFAs | 0.98 | 0.97 | 0.99 | 0.98 | 0.98 | 0.98 | 0.98 | 0.99 |
|  | Change in total n-3 PUFAs | 0.87 | 0.86 | 0.87 | 0.87 | 0.86 | 0.87 | 0.87 | 0.87 |
|  | Change in n-6:n-3 ratio | 1.18 | 1.19 | 1.18 | 1.17 | 1.18 | 1.17 | 1.18 | 1.18 |
|  | Change in DHA % total fatty acids | 0.96 | 0.95 | 0.96 | 0.96 | 0.96 | 0.97 | 0.96 | 0.96 |
| **Generalised anxiety disorder** | Change in total n-6 PUFAs | 0.83 | 0.82 | 0.83 | 0.82 | 0.83 | 0.83 | 0.83 | 0.83 |
|  | Change in total n-3 PUFAs | 0.88 | 0.87 | 0.87 | 0.88 | 0.88 | 0.88 | 0.88 | 0.88 |
|  | Change in n-6:n-3 ratio | 1.02 | 1.03 | 1.03 | 1.01 | 1.02 | 1.02 | 1.02 | 1.02 |
|  | Change in DHA % total fatty acids | 0.97 | 0.97 | 0.97 | 0.98 | 0.97 | 0.97 | 0.97 | 0.97 |

AUDIT: Alcohol Use Disorders Identification Test; CI: confidence interval; DHA: docosahexaenoic acid; n-6: omega-6; n-3: omega-3; PUFA: polyunsaturated fatty acid.
^a^ Regular cannabis use at age 17 was defined as using cannabis more than once monthly.

PUFA measures were converted to z-scores such that the odds ratios may be interpreted as per standard deviation increase in the PUFA measure.

**Supplementary Figure 1: Participant flow diagram depicting sample derivation**

14,901 participants

alive at 1 year

9958 invited to
age 24 clinic

Attended one or both clinics, *n*=6087

9919 invited to
age 17 clinic

Attended and clinic data available, *n*=5215

Attended and clinic data available, *n*=4019

PUFA data available

at age 17, *n*=3166
(1 outlier excluded)

Outcomes data available
at age 24

Psychotic disorder, *n*=3889

Moderate/severe

depressive disorder, *n*=3966

GAD, *n*=3957

PUFA data available

at age 24, *n*=3257

Outcomes data available
at age 17

Psychotic disorder, *n*=4718
Moderate/severe

depressive disorder, *n*=4563

GAD, *n*=4563

PUFA and outcomes data available at age 17

Psychotic disorder, *n*=2987

Moderate/severe

depressive disorder, *n*=2897

GAD, *n*=2897

PUFA and outcomes data available at age 24

Psychotic disorder, *n*=3164

Moderate/severe

depressive disorder, *n*=3216

GAD, *n*=3208

Exposures and

covariates imputed

Psychotic disorder, *n*=4718
Moderate/severe

depressive disorder, *n*=4563

GAD, *n*=4563

Exposures and

covariates imputed

Psychotic disorder, *n*=3889

Moderate/severe

depressive disorder, *n*=3966

GAD, *n*=3957

GAD: generalised anxiety disorder; PUFA: polyunsaturated fatty acids
